# Naturally derived indole alkaloids targeting regulated cell death (RCD) for cancer therapy: from molecular mechanisms to potential therapeutic targets

**DOI:** 10.1186/s13045-022-01350-z

**Published:** 2022-09-14

**Authors:** Rui Qin, Feng-Ming You, Qian Zhao, Xin Xie, Cheng Peng, Gu Zhan, Bo Han

**Affiliations:** 1 State Key Laboratory of Southwestern Chinese Medicine Resources, Hospital of Chengdu University of Traditional Chinese Medicine, School of Pharmacy, Chengdu University of Traditional Chinese Medicine, Chengdu, 611137 China; 2 School of Basic Medical Sciences, Chengdu University of Traditional Chinese Medicine, Chengdu, 611137 China; 3 College of Medical Technology, Chengdu University of Traditional Chinese Medicine, Chengdu, 611137 China

**Keywords:** Cancer, Indole alkaloids, Regulated cell death (RCD), Apoptosis, Autophagy, Ferroptosis, Mitotic catastrophe, Necroptosis, Anoikis, Target therapy

## Abstract

Regulated cell death (RCD) is a critical and active process that is controlled by specific signal transduction pathways and can be regulated by genetic signals or drug interventions. Meanwhile, RCD is closely related to the occurrence and therapy of multiple human cancers. Generally, RCD subroutines are the key signals of tumorigenesis, which are contributed to our better understanding of cancer pathogenesis and therapeutics. Indole alkaloids derived from natural sources are well defined for their outstanding biological and pharmacological properties, like vincristine, vinblastine, staurosporine, indirubin, and 3,3′-diindolylmethane, which are currently used in the clinic or under clinical assessment. Moreover, such compounds play a significant role in discovering novel anticancer agents. Thus, here we systemically summarized recent advances in indole alkaloids as anticancer agents by targeting different RCD subroutines, including the classical apoptosis and autophagic cell death signaling pathways as well as the crucial signaling pathways of other RCD subroutines, such as ferroptosis, mitotic catastrophe, necroptosis, and anoikis, in cancer. Moreover, we further discussed the cross talk between different RCD subroutines mediated by indole alkaloids and the combined strategies of multiple agents (e.g., 3,10-dibromofascaplysin combined with olaparib) to exhibit therapeutic potential against various cancers by regulating RCD subroutines. In short, the information provided in this review on the regulation of cell death by indole alkaloids against different targets is expected to be beneficial for the design of novel molecules with greater targeting and biological properties, thereby facilitating the development of new strategies for cancer therapy.

**Graphic abstract:**

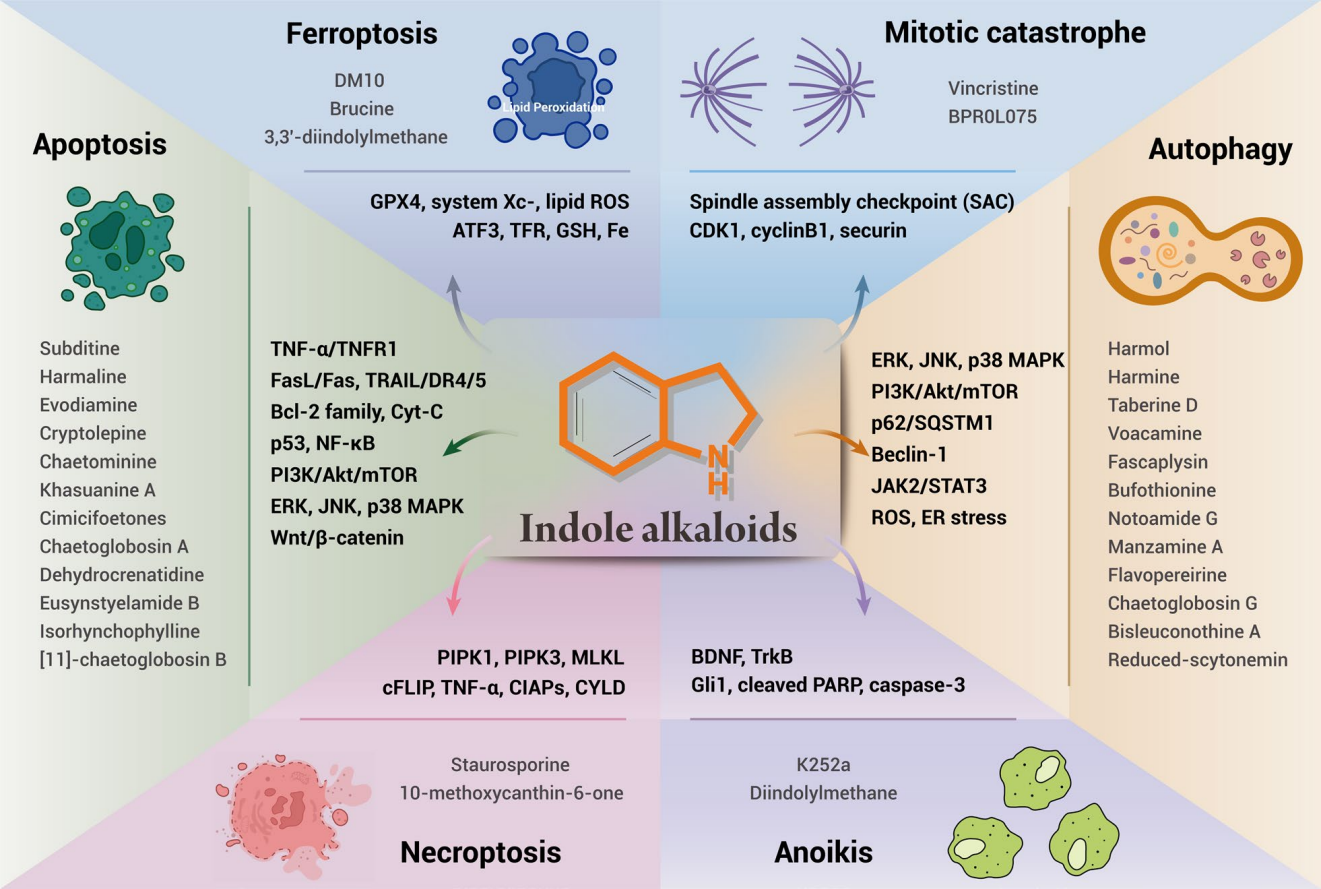

## Background

Based on statistics from the World Health Organization (WHO) in 2019, cancer is one of the top two causes of human death in 127 of the 183 countries [1]. With the current increasing trends of major cancer types, cancer may overtake cardiovascular diseases as the leading cause of early death in most countries this century [1, 2]. Chemotherapy, surgery, and radiotherapy have become the three pillars of fighting cancer, but with the aggravation of drug resistance and the emergence of intolerable side effects, developing novel and feasible anticancer drugs and alternative therapies has become the most urgent task. Targeted therapy is a new therapeutic method that can specifically target cancer cells without harming normal cells, thus possessing the characteristics of high efficacy and low toxicity [3, 4]. Over the past decade, the Nomenclature Committee on Cell Death (NCCD) has established guidelines for the definition and explanation of cell death from the viewpoints of morphological, biochemical, and functional, respectively [5]. Based on functional aspects, cell death modalities can be classified into two types: accidental cell death (ACD) and regulated cell death (RCD). ACD is usually triggered by some uncontrollable factors, including chemical, physical, or mechanical stress. In contrast, RCD is a cellular process that is controlled by precise signal transduction pathways and molecularly defined effector mechanisms, as well as regulated by pharmacological or genetic interventions [5, 6]. RCD plays a critical role in the homeostasis of tissue, and disturbances in this process have been associated with a multitude of diseases, including neurodegenerative diseases, immunological disorders, and cancer [7]. Since RCD is executed by specific proteins, pharmacological targets of these modulators can be utilized in the treatment of cancer. Different subroutines of RCD processes can distinctively affect tumor progression and response to therapeutics [7–9].

**Fig. 1:**
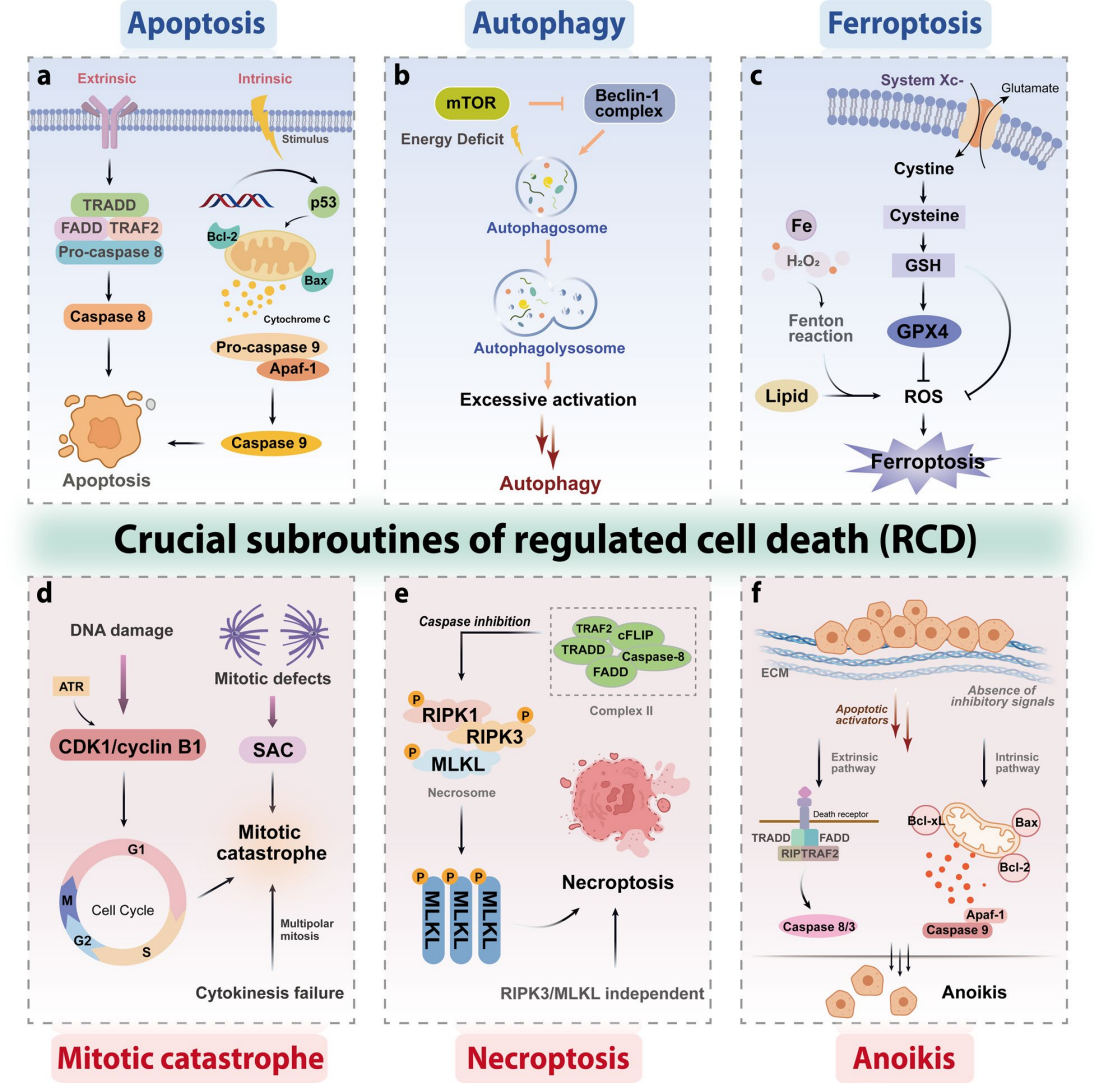
Core molecular mechanism of apoptosis, autophagy, ferroptosis, mitotic catastrophe, necroptosis, and anoikis. **a** There are two well-known signal transduction cascades regulating cell apoptosis: the extrinsic and intrinsic pathways. The extrinsic pathway is activated by death receptors and death ligands, while the intrinsic pathway is initiated by cellular stress-mediated mitochondria dysfunction. **b** The formation of the autophagosome depends on the formation of a complex incorporating Beclin-1, which is regulated by mTOR. Moreover, various proteins and signaling molecules (AMPK, PI3K, p62, etc.) are involved in the regulation of autophagy. **c** Ferroptosis is a type of regulated cell death that is induced by the iron-dependent accumulation of lipid reactive oxygen species (ROS) and lipid peroxidation, the inhibition of cystine/glutamate antiporter, and the loss of activity of glutathione peroxidase 4 (GPX4). **d** The cyclin-dependent kinase 1 (CDK1)/cyclin B1 complex is an important component of mitotic catastrophe and can promote cell cycle transition from G2 phase to M phase. Deoxyribonucleic acid (DNA) damage, mitotic defects, and cytokinesis failure are the three key factors leading to mitotic catastrophes. **e** Necroptosis is a form of regulated necrotic cell death stimulated by tumor necrosis factor-α (TNF-α). After TNFα binds to the receptor, tumor necrosis factor receptor 1 (TNFR1) recruits TNFRSF1A associated via death domain (TRADD), Fas associated via death domain (FADD), receptor-interacting serine/threonine kinase protein (RIPK) 1, TNF receptor-associated factor 2 (TRAF2) and other proteins to form complex I, and then RIPK1 is deubiquitinated to promote the transformation of complex I to complex II. When caspase-8 is inhibited, mixed lineage kinase domain-like protein (MLKL), RIPK1, and RIPK3 are recruited to form necrosome through phosphorylation, which eventually triggers necroptosis. **f** Anoikis induces cell death through conventional apoptotic pathways. B cell lymphoma 2 (Bcl-2) related proteins are widely involved in anoikis regulation, and multiple protein kinases are involved in the signal transduction of anoikis. When cells detach from the extracellular matrix (ECM), pro-survival signals cannot be activated, but the death receptors and mitochondrial apoptotic pathways are activated to prevent adherent-independent cell growth and attachment, and finally activate anoikis to induce cell death

Remarkably, apoptosis, autophagy, ferroptosis, mitotic catastrophe, necroptosis, and anoikis are important subroutines of RCD, each of which has unique molecular mechanisms and has critical roles in cancer advancement and targeted therapy (Fig. 1). Indole alkaloids are a diverse class of natural products with complex chemical structures, which can be separated from a large number of natural sources and possess a multitude of pharmacological activities, such as antibacterial, antimalarial, anti-inflammatory, antiviral, and anticancer properties [10, 11]. Through literature investigation, it was found that indole alkaloids can regulate cell death by targeting the death mechanisms and related signaling pathways, thus exerting a powerful anticancer effect [12]. So far, numerous natural and synthetic indole derivatives have been discovered as promising anticancer agents used in the clinic or clinical evaluations, such as vincristine, vinblastine, indirubin, indole-3-carbinol, and harmine, indicating its prominent place in anticancer drugs development.

In this review, we will concentrate our efforts on various natural sources of indole alkaloids that regulate tumor cell apoptosis, autophagy, ferroptosis, mitotic catastrophe, necroptosis, and anoikis, through the modulation of multiple cellular signaling pathways, with the aim of providing a basis for the subsequent studies on the mechanism of cell death induced by natural anticancer drugs, thereby developing more selective natural products and their derivatives, expanding the existing compound library, and assisting the improvement in existing therapeutic strategies. Further, we will also briefly discuss the combined strategies of multiple indole alkaloids along with chemotherapeutics by regulating RCD subroutines in cancer therapy. Our further understanding of the role of indole alkaloids in targeting regulated cell death is expected to provide prospective strategies for cancer therapy.

## Indole alkaloids

Natural products are well considered the “treasure trove of small molecule drugs.” More than 60% of the antineoplastic drugs approved by the Food and Drug Administration (FDA) are found to be of natural sources (e.g., paclitaxel, topotecan, or vincristine) and can be used in their monomeric form or as lead compounds with simple modifications [13, 14]. Moreover, in other fields, the influence of natural product structures is quite significant, and the development of anti-infective drugs also relies on natural products and their structures, which shows that the exploitation of natural products and/or synthetic derivatives of their new structures to discover and develop the final drug entities, is still practical as well as popular [13, 15]. Natural products, including terpenoids, flavonoids, quinones, lignans, glycosides, coumarins, chromones, and alkaloids, demonstrate the structural diversity of plant-derived compounds, thus making natural products as effective templates for new therapeutic approaches and new drugs, and still the most popular choice in the field of drug development [16, 17]. Among the natural resources, plants remain the largest source for discovering new compounds, followed by animals, marine organisms, and terrestrial microorganisms [15]. Accordingly, some studies on natural products have been successful so far. Artemisinin, as a natural sesquiterpene lactone, is widely used in malaria prevention and treatment [18]; paclitaxel, as a natural antitumor drug, can act on microtubules and block the division of deoxyribonucleic acid (DNA), and is mostly used clinically for the treatment of various solid tumors such as lung cancer, breast cancer, and ovarian cancer; colchicine, an important alkaloid originally found in *Liliaceae*, has a selective anti-inflammatory effect on acute gouty arthritis [19]. Additionally, camptothecin is an alkaloid derived from *Camptotheca acuminata* Decne with superior anticancer activity as a topoisomerase I inhibitor that inhibits DNA replication of tumor cells. At present, a series of semisynthetic and fully synthetic derivatives of camptothecin (e.g., irinotecan, topotecan, rubitecan, etc.) have emerged and entered the clinical applications or clinical trials [20]. The discovery of the natural product camptothecin provides a lead structure for a class of classical and promising anticancer drugs.

**Fig. 2:**
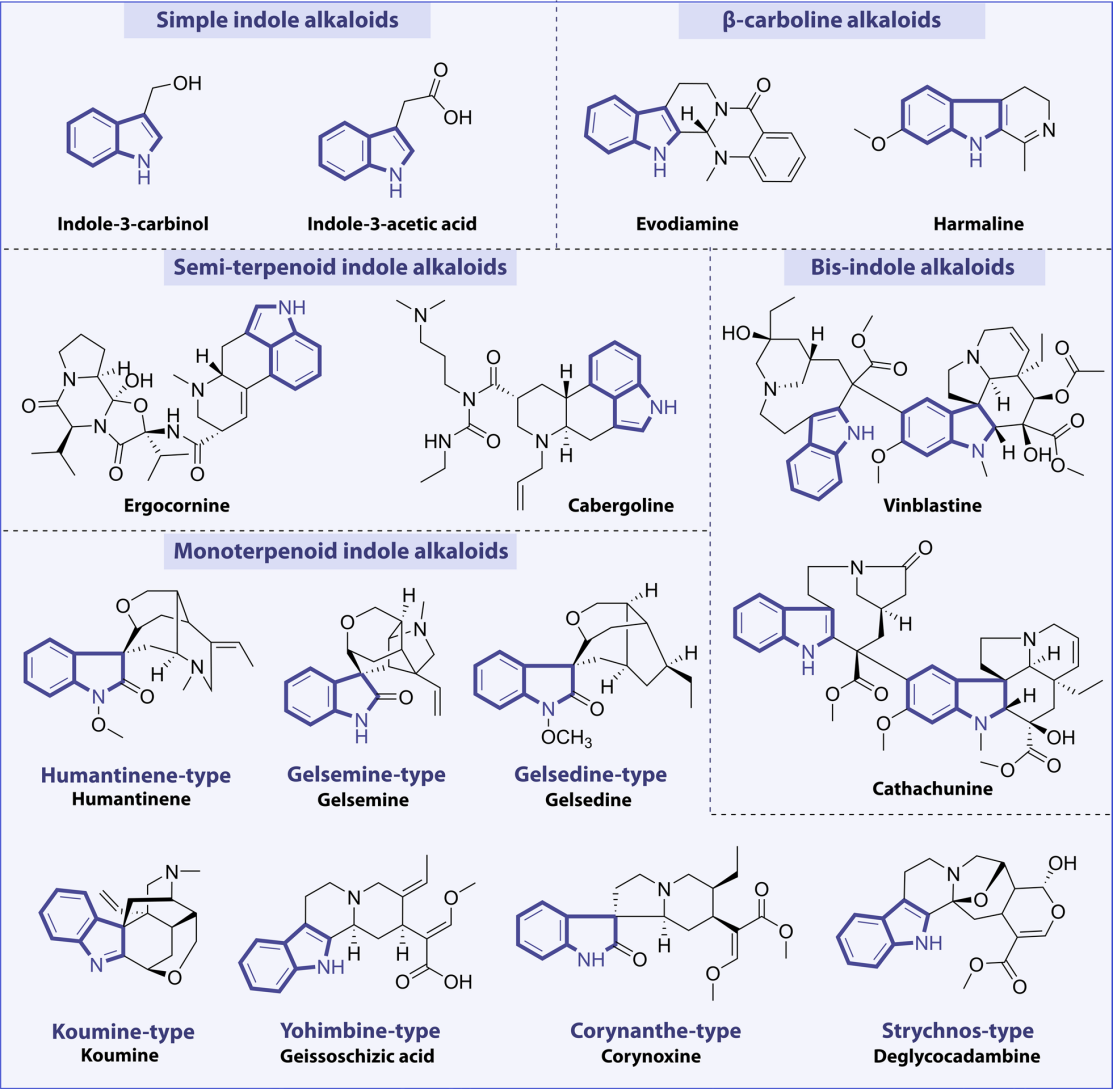
Classification of indole alkaloids and their representative compounds

**Fig. 3:**
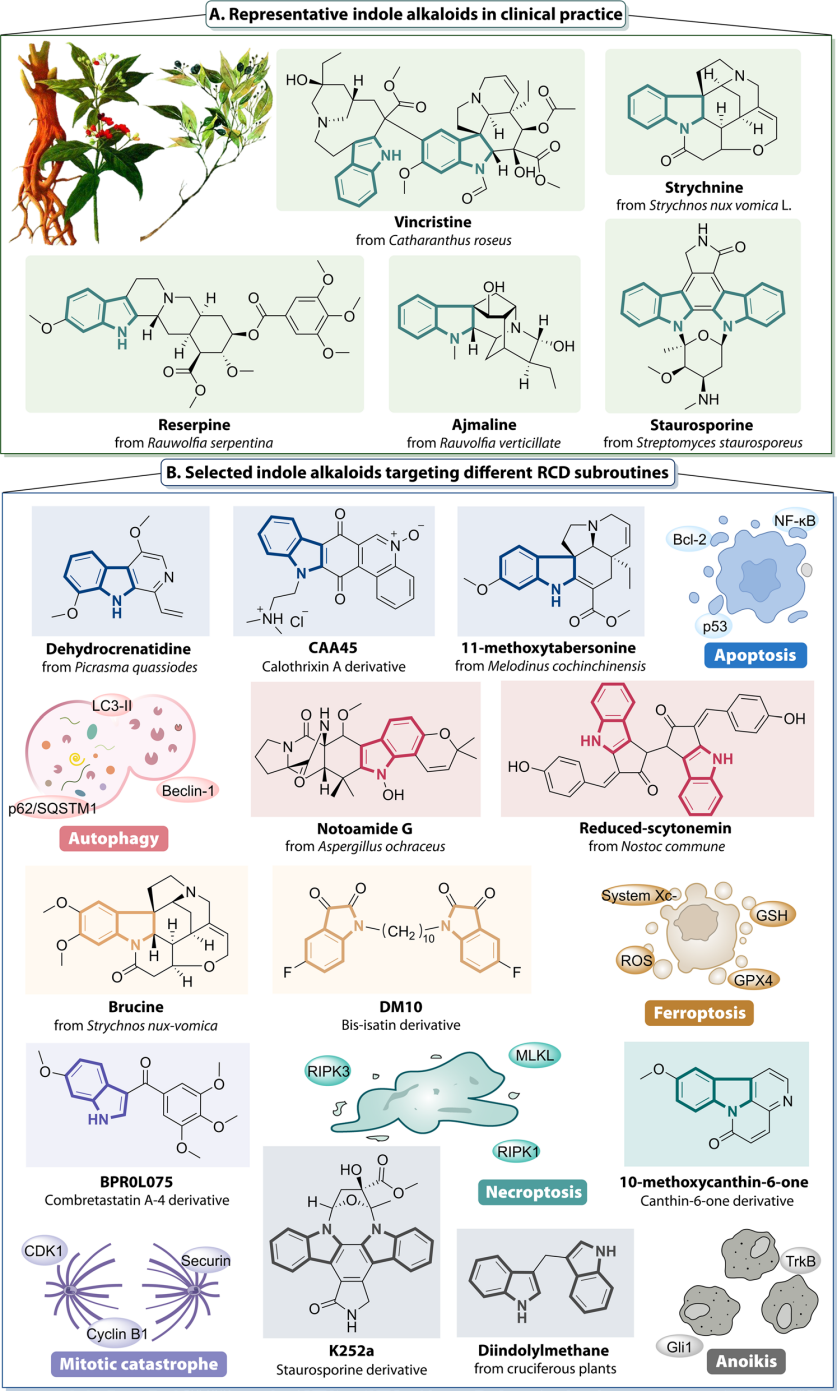
Representative indole alkaloids in clinical and preclinical studies. **A** Natural indole alkaloids are an important source of lead compounds, and the above chart shows representative natural indole alkaloids that have been used in clinic. **B** Indole alkaloids and their derivatives regulate tumor cell apoptosis, autophagy, ferroptosis, mitotic catastrophe, necroptosis, and anoikis, through a variety of cellular signaling pathways, which are currently in preclinical studies and are expected to provide promising strategies for cancer therapy

Alkaloids are a class of basic nitrogenous organic compounds ubiquitously found in nature, with remarkable bioactivity profiles, often combined with acid compounds in the form of alkaloid salts widely exist in *Papaveraceae*, *Menispermaceae*, *Ranunculaceae*, *Solanaceae*, *Apocynaceae*, *Rutaceae*, *Berberidaceae*, *Leguminosae*, *Polygonaceae*, *Rubiaceae*, etc. [21]. Alkaloids are important active components of many medicinal plants of natural origin, which can be divided into many subclasses according to their structures, mainly including indoles, pyridines, tropanes, amphetamines, quinolines, isoquinolines, pyrrolidines, steroids, and terpenoids [22]. Among them, indole alkaloids are the typical class of alkaloids with wide varieties, complex structures, and the largest number of compounds [11]. They are also one of the most studied natural products nowadays. Indole alkaloids are heterocyclic natural products formed by the fusion of a benzene ring and a pyrrole ring. The existence of the nitrogen atom leads to the basic characteristics of indole alkaloids, which make them extensive pharmacological activities. According to the structure types and skeleton characteristics of indole alkaloids, they can be classified into simple indole alkaloids, β-carboline alkaloids, semi-terpenoid indole alkaloids, bisindole alkaloids, and monoterpenoid indole alkaloids. Among them, monoterpenoid indole alkaloids represent the largest number of compounds with relatively complex classification, which can be classified according to their chemical structures, including humantenine, gelsemine, gelsedine, koumine, yohimbine, corynanthe, and strychnos types [10, 23]. Several representative compounds are selected and listed in Fig. 2. Indole alkaloids are currently a hotspot of research for pharmacologists due to their high market share and multiple physiological activities. These compounds have a broad range of biological activities, such as anxiolytic, anticonvulsant, antiviral, anti-inflammatory, anti-fibrosis, antiparasitic, antibacterial, anti-arrhythmic, and antitumor properties [10, 24, 25]. Particularly, most of them are isolated from marine organisms such as fungi, bacteria, mollusks, sponges, and algae. By systematically analyzing the anticancer mechanism and targets of these marine-derived indole alkaloids, it would be able to obtain lead compounds for the development of new drugs [26]. As an important source of lead compounds, many clinical agents have been obtained from natural indole products. Physostigmine is the simplest indole alkaloid extracted from the Calabar Bean, which can inhibit cholinesterase activity and is mainly used in the treatment of glaucoma [27]. Reserpine, derived from the species *Rauvolfia serpentine*, can be used as an antihypertensive agent and tranquilizer. Strychnine, clinically used as strychnine nitrate, causes intense excitement and convulsion of central and spinal nerves and belongs as a central nervous stimulant. Another alkaloid, ajmaline, is isolated from the plant *Rauvolfia verticillata* with anti-hypertension, sedative, and anti-arrhythmic effects [28]. Yohimbine, derived from the dried bark of *Corynanthe Yohimbe*, has been used to treat erectile dysfunction. Ergometrine is a uterotonic agent that is used to prevent and treat postpartum hemorrhage [29]. In addition, indole alkaloids are recognized as important sources of anticancer drugs. Vinca alkaloids, derived from the species *Catharanthus roseus*, structurally belong to bis-indole alkaloids and are well known for their excellent antitumor activities [30]. The natural products of this group, such as vinblastine, vincristine, vindesine, and its structural modification compounds, vinorelbine, currently have become commercial drugs and have been used as first-line drugs in the clinical treatment of cancer. All of these drugs act relatively well, but their use is limited by serious side effects, including neurotoxicity and bone marrow suppression [30, 31]. Perhaps the discovery of a novel method of drug delivery through liposome-encapsulated drugs, nano-preparations, and polymer-packaged drugs could be found to reduce the toxicity and improve the efficacy of vinca alkaloids [32]. Consequently, it is of great significance to design and synthesize novel vinca alkaloids with similar efficacy but low toxicity. Moreover, β-carboline alkaloids are an important class of indole alkaloids consisting of tricyclic pyrido-[3,4-b]indole ring, with the widest distribution in nature, mainly extracted from the seeds of *Peganum harmala* [33, 34]. Some β-carboline alkaloids, such as harmaline, harmine, subditine, and flavopereirine, exhibited remarkably antitumor properties, which has inspired researchers for further investigation [35]. Among the various agents or methods for the treatment of oncological diseases, the treatment with natural indole alkaloids has shown promising results because of their superior efficiency and availability, as well as low side effects (Fig. 3).

Furthermore, it can be clearly observed that several well-known drugs on the market are based on indole frameworks. Some representative drugs, like osimertinib, the first and only targeted drug, are approved for the treatment of non-small cell lung cancer (NSCLC) with epidermal growth factor receptor (EGFR) T790M mutation [36]. Sunitinib, an oral multitarget tyrosine kinase inhibitor, is used to treat metastatic renal cell carcinoma and gastrointestinal stromal tumor [37]. It is also the first drug approved for the simultaneous treatment of two types of cancer. Leuprorelin acetate, an indole gonadotropin-releasing hormone receptor (GnRHR) agonist, is primarily used to treat prostate cancer and breast cancer [38]. Therefore, synthetic compounds oriented by the indole ring, the dominant scaffold, could be excellent sources of new drug candidates, particularly in the field of anticancer therapeutics [39–42]. Overall, these inspiring discoveries would shed light on exploiting more natural indole alkaloids and their derivatives as candidate small-molecule agents to target and regulate tumor cell apoptosis, autophagy, ferroptosis, mitotic catastrophe, necroptosis, and anoikis through different signaling pathways, so as to provide help for future cancer therapy.

## Targeting apoptotic signaling pathways with indole alkaloids in cancer

Apoptosis is the most well-characterized form of regulated cell death, which is finely regulated at the genetic level and is of vital importance in maintaining the stability of the tissue homeostasis through the orderly elimination of damaged cells, and its disorders may lead to cancer [43]. From the morphological point of view, apoptosis is accompanied by some typical properties, such as cytoplasmic cell shrinkage and floating, budding of the plasma membrane, chromatin condensation, DNA fragmentation, and production of apoptotic bodies [44, 45]. Now it is generally accepted that abnormalities in the processes of apoptosis are the main cause of carcinogenesis. Cancer cells can escape apoptosis in various cell death, and inhibition of apoptotic signals may play a very important role in tumor formation [44]. Therefore, inducing tumor cells to regain their apoptotic ability is an important means to improve the antitumor effect during cancer treatment. Moreover, a comprehensive understanding of the mechanisms of apoptosis in different cell types is also essential for the development of potential cancer therapies.

**Fig. 4:**
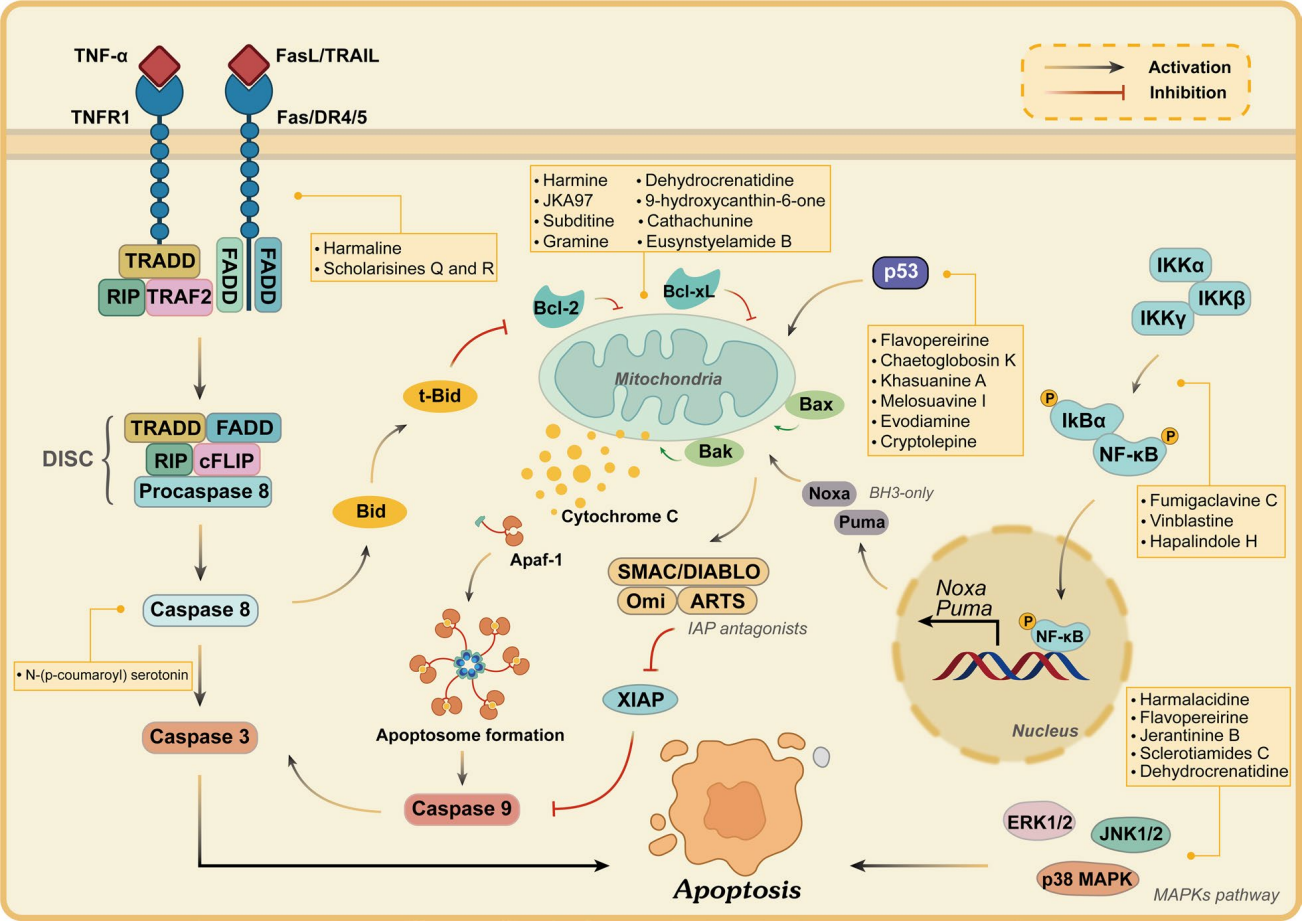
Core apoptosis signaling pathway in cancer. Apoptosis is activated by two pathways, the extrinsic and intrinsic pathways. The extrinsic pathway is triggered by death receptors such as TNFR1, Fas, and death receptor (DR) 4/5 by their related ligands TNF-α, Fas ligand (FasL), and TNF-related apoptosis-inducing ligand (TRAIL). Ligand binding to these receptors leads to the recruitment of FADD and TRADD, and then forms a complex called death-inducing signaling complex (DISC), which in turn results in the cleavage of procaspase-8 and sends a signal to activate caspase-8, thus activating caspase-3, and ultimately leads to apoptosis. The intrinsic pathway is stimulated by cellular stress, leading to p53 and BH3 only proteins activation, which in turn induces Bak/Bax oligomerization and permeabilization of the mitochondria, and ultimately promotes the release of cytochrome C (Cyt-C). Cyt-C forms a complex with apoptotic protease activating factor 1 (Apaf-1) and procaspase-9 and then activates caspase-9. Caspase-9 triggers the caspase-3 activation and induces apoptotic cell death. Besides, the inhibitor of apoptosis protein (IAP) family negatively regulates caspase activation and can be inhibited by second mitochondria-derived activator of caspase (SMAC)/direct IAP-binding protein with low pI (DIABLO), Omi, and apoptosis-related protein in the TGF-β signaling pathway (ARTS). Under certain conditions, cross talk from the extrinsic pathway via caspase-8-mediated truncation of Bid to t-Bid can also cause mitochondrial permeabilization. Additionally, the mitogen-activated protein kinase (MAPK) and nuclear factor kappa-B (NF-κB) pathways also play essential roles in regulating apoptotic cell death

There are two distinct mechanisms regulating cell apoptosis: the extrinsic pathway stimulated by the binding of cell surface death receptors with ligands; and the intrinsic pathway initiated by the activation of intracellular signals from mitochondria [46]. The extrinsic apoptotic pathway begins with the binding of death ligands, such as tumor necrosis factor-α (TNF-α), Fas ligand (FasL), and TNF-related apoptosis-inducing ligand (TRAIL), to their corresponding receptors such as tumor necrosis factor receptor 1 (TNFR1), Fas, and death receptor (DR) 4/5 [47, 48]. Subsequently, the interaction of death receptors with ligands recruits downstream molecules via the TNF receptor-associated death domain (TRADD) and Fas-associated death domain (FADD), which then bind to pro-caspase-8 by the death effector domain (DED) to form the death-inducing signaling complex (DISC). DISC activates the apoptosis initiator protein caspase-8 through the cleavage of pro-caspase-8 and eventually leads to the execution phase of apoptotic cell death [9, 49]. In addition, activated caspase-8 also triggers mitochondrial damage through cleavage of the BH3-only protein Bid, thereby activating the intrinsic apoptotic pathway. Hypoxia, viral infections, growth factor deprivation, Ca^2+^ overload, oxidative stress, chemotherapeutic agents, and another stimulus all trigger activation of the intrinsic pathway [50]. Changes in mitochondrial outer membrane permeability (MOMP) mark the activation of the mitochondria apoptosis pathway, and in addition, the intrinsic pathway is tightly regulated by the B cell lymphoma 2 (Bcl-2) family proteins, including the pro-apoptotic proteins (such as Bax and Bak), the anti-apoptotic proteins [such as Bcl-2, B cell lymphoma-extra-large (Bcl-xL), and myeloid cell leukemia-1 (Mcl-1)], and the Bcl-2 homology domain 3 (BH3)-only proteins (such as Bid, Bim, Noxa, and Puma) [51]. These proteins regulate the mitochondrial membrane to secrete cytochrome C (Cyt-C), and the interaction between Cyt-C, apoptotic protease activating factor 1 (Apaf-1), and caspase-9 forms a multimeric complex called the apoptosome, which in turn induces the activation of caspase-9 and caspase-3, thereby eliciting apoptosis of cancer cells [52]. Besides, the suppression of caspase-9 is also mediated by an X-linked inhibitor of apoptosis protein (XIAP), which is degraded by IAP antagonists, the second mitochondria-derived activator of caspase (SMAC)/direct IAP-binding protein with low pI (DIABLO), Omi, and apoptosis-related protein in the TGF-β signaling pathway (ARTS) during cell death [53]. Statistically, the Bcl-2 family proteins largely control this process (Fig. 4).

Apoptosis is assumed to be one of the pivotal mechanisms in cancer therapy and has become an effective target for the discovery and development of novel anticancer drugs. Natural products have long been considered important sources of new antitumor drugs, among which indole alkaloids can be isolated from various natural sources and have been shown to possess remarkable pharmacological properties. Studies have revealed that several indole alkaloids induce apoptosis via different mechanisms and pathways, thus exerting antineoplastic effects [54, 55]. Next, we will discuss the antitumor activity of indole alkaloids in cancer in terms of apoptosis-related signaling pathways and targets, and the essential pathways and targets of apoptosis include death receptors and their ligands, Bcl-2 family, cytochrome c (Cyt-C), p53, nuclear factor kappa-B (NF-κB) pathway, phosphatidylinositol 3 kinase (PI3K)-protein kinase B (Akt)-mammalian target of rapamycin (mTOR) pathway, mitogen-activated protein kinase (MAPK) pathway, and so on.

### Targeting death receptors and their ligands

Death receptors and their ligands are the central components that mediate the extrinsic apoptosis of cells. When cell surface death receptors (such as TNFR1, Fas, and DR4/5) bind to extracellular ligands (such as TNF-α, FasL, and TRAIL), it will result in DISC formation, trigger caspase cascade activation, ultimately inducing apoptosis [48]. Recently, research gradually found that indole alkaloids derived from natural sources can regulate death receptors and their ligands, thus leading to the induction of apoptosis in cancer (Table 1).

**Table 1:** Indole alkaloids targeting death receptors and their ligands, Bcl-2 family and Cyt-C, in cancer

Compound	Structure	Source	Target	Mechanism in RCD	Cancer cell line	Tumor type	Refs.
Harmaline		*Peganum harmala*	Fas/FasL↑ caspase-8/-3↑	Induce apoptosis	SGC-7901 (IC_50_ = 4.08 ± 0.89 μM)	Gastric cancer	[56]
Scholarisines Q and R		*Alstonia scholaris*	TNF-α, caspase-3↑	Induce apoptosis	GSC-3#, GSC-12#, GSC-18# (IC_50_ = 15–25 μM)	Glioblastoma multiform	[57]
*N*-(*p*-coumaroyl) serotonin		*Centaurea vlachorum*	Caspase-8↑	Induce apoptosis	U251MG (IC_50_ = 77 μM); T98G (IC_50_ = 68 μM)	Glioblastoma	[59]
Harmine		*Peganum harmala*	COX-2, Bcl-2, MMP-2↓ Bax↑	Induce apoptosis	BGC-823 (IC_50_ = 3.63 ± 0.54 μM); SGC-7901 (IC_50_ = 2.92 ± 0.34 μM)	Gastric cancer	[65]
			TAZ, Bcl-2↓ Bax↑		MDA-MB-231 and MCF-7	Breast cancer	[66]
JKA97		Harmine derivative	Bax, Cyt-C↑	Induce apoptosis	HCT116	Colon cancer	[67]
			p21↑		MCF-7 (IC_50_ = 19.0 ± 0.7 μM); MDA-MB-468 (IC_50_ = 6.6 ± 0.5 μM); MCF-7 p53KD (IC_50_ = 10.3 ± 2.7 μM)	Breast cancer	[68]
Subditine		*Nauclea subdita*	Bcl-2, Bcl-xL↓ Cyt-C, p53↑	Induce apoptosis	LNCaP (IC_50_ = 12.24 ± 0.19 μM); PC-3 (IC_50_ = 13.97 ± 0.32 μM)	Prostate cancer	[69]
Dehydrocrenatidine		*Picrasma quassioides*	Bax↑ Bcl-2↓	Induce apoptosis	HepG2 (IC_50_ = 3.50 ± 0.29 μM); Hep3B (IC_50_ = 5.87 ± 0.48 μM)	Hepatocellular carcinoma	[70]
9-Hydroxycanthin-6-one		*Ailanthus altissima*	Caspase-3/8/9↑	Induce apoptosis	A2780 (IC_50_ = 10.62 μM), SKOV3 (IC_50_ = 34.7 μM), and OVCAR-3 (IC_50_ = 42.13 μM)	Ovarian cancer	[71]
Evodiamine		*Evodia rutaecarpa*	Cyt-C↑	Induce apoptosis	A549, H1299, A431	Lung cancer	[73]
			Mcl-1↓	Induce apoptosis	253 J (IC_50_ = 1.90 ± 0.31 μM); T24 (IC_50_ = 2.14 ± 0.26 μM)	Bladder cancer	[76]
Vinorelbine		Vinca alkaloid	Bax↑ Bcl-2, Bcl-xL↓	Induce apoptosis	H1975 (IC_50_ = 0.008 ± 0.001 μM); HepG2 (IC_50_ = 0.11 ± 0.01 μM); and HCT116 (IC_50_ = 0.0149 ± 0.002 μM)	Lung cancer; liver cancer; colorectal cancer	[77]
Vincamine		*Vinca minor*	Caspase-3, Cyt-C↑	Induce apoptosis	A549 (IC_50_ = 309.7 μM)	Lung cancer	[78]
Gramine		Barley and silver maple	Bcl-2↓ Bax, Cyt-C, apaf-1↑	Induce apoptosis	7,12-Dimethylbenz[a]anthracene (DMBA)-induced hamster buccal pouch	Oral squamous cell carcinoma	[82]
Gramine derivative (16 h)		Gramine derivative	Bax, PARP↑ Bcl-2↓	Induce apoptosis	MGC803 (IC_50_ = 3.74 μM)	Gastric cancer	[83]
Indole-3-carbinol		Cruciferous vegetables	ROS, Bax↑ Bcl-2, Bcl-xL↓	Induce apoptosis	H1299	Lung cancer	[84]
Cathachunine		*Catharanthus roseus*	Bcl-2↓ Bax, Cyt-C↑	Induce apoptosis	HL60 (IC_50_ = 9.1 ± 0.7 μM); K562 (IC_50_ = 9.3 ± 1.8 μM)	Leukemia	[85]
(3′R)-hydroxytaberanelegantine C		*Tabernaemontana elegans*	Bcl-2, XIAP↓	Induce apoptosis	HCT116 (IC_50_ = 3.20 μM); SW620 (IC_50_ = 3.49 μM); HepG2 (IC_50_ = 3 μM)	Colorectal cancer, colon cancer, liver cancer	[86]
Cimicifoetones		*Cimicifuga foetida*	Caspase-3/-8/-9↑ Bcl-2, Bcl-xL↓ Bax↑	Induce apoptosis	HL-60 (IC_50_ = 1.36 ± 0.09 μM)	Leukemia	[87]
Staurosporine		*Streptomyces staurosporeus*	Bcl-2, Bad↓ Bax↑	Induce apoptosis	PaTu 8988t, Panc-1	Pancreatic cancer	[88]
2,2-Bis(6-bromo-3-indolyl) ethylamine		*Didemnum candidum* and the New Caledonian sponge *Orina*	Bcl-2, Bcl-xL↓ Bax↑	Induce apoptosis	U937	Leukemia	[89]
Eusynstyelamide B		*Didemnum candidum*	Cleaved PARP, caspase-3↑	Induce apoptosis	MDA-MB-231 (IC_50_ = 5.0 μM)	Breast cancer	[90]
Chetomin		*Chaetomium cochliodes*	Bcl-2↓ Bax, Cyt-C↑ PI3K/mTOR↓	Induce apoptosis	MDA-MB-231; MDA-MB-468	Triple-negative breast cancer	[91]
Chaetoglobosin A		*Penicillium aquamarinium*	Caspase-3↑	Induce apoptosis	MEC-1	Chronic lymphocytic leukemia	[92]
[11]-chaetoglobosin B		*Pseudeurotium bakeri* P1-1–1	Bax, Cyt-C, PARP↑ Bcl-2↓	Induce apoptosis	MCF-7 (IC_50_ = 6.2 ± 1.1 μM); A427 (IC_50_ = 4.7 ± 1.0 μM)	Breast cancer; lung cancer	[93]
Chaetominine		*Aspergillus fumigatus*	ROS, Bax↑ Bcl-2↓	Induce apoptosis	K562 (IC_50_ = 33.7 ± 0.2 nM); K562/Adr (IC_50_ = 47.9 ± 1.1 nM)	Leukemia	[94]
Reserpine		*Rauwolfia serpentina*	TGF-β↓ Bax, Cyt-C, Apaf-1↑	Induce apoptosis	DMBA-induced hamster buccal pouch carcinogenesis model	Oral squamous cell carcinoma	[96]
Brucine		*Strychnos nux vomica* L	Caspase-3, Bax↑ Bcl-2↓	Induce apoptosis	Rats	Hepatocellular carcinoma	[98]
Jerantinine B		*Tabernaemontana corymbosa*	Bcl-2, Mcl-1↓	Induce apoptosis	HCT-116, A549, MCF-7 (GI_50_ = 0.7–0.9 μM); MIA PaCa-2 (GI_50_ ≈ 0.25 μM)	Colorectal cancer; gastric cancer; breast cancer; pancreatic cancer	[99]
Isorhynchophylline		*Uncaria rhynchophylla*	Bcl-2, Bcl-xL↓	Induce apoptosis	HepG2 (IC_50_ = 130 μM)	Hepatocellular carcinoma	[100]
Bufothionine		Toad Bufo bufo gargarizans Cantor	Bcl-2↓ Bax↑	Induce apoptosis	MKN28 and AGS cells	Gastric cancer	[101]
CAA45		Calothrixin A derivative	Bcl-2↓ Bax, Bad, Cyt-C↑	Induce apoptosis	A549 (IC_50_ = 110 nM); NCI-H1650 (IC_50_ = 230 nM)	Lung cancer	[102]
(*E*)-1-benzyl-5-bromo-3-{[1-(2,5-dimethoxyphenyl)-9*H*-pyrido[3,4-b]indol-3-yl]methylene}indolin-2-one		β-carboline derivative	Bax, PARP↑ Bcl-2↓	Induce apoptosis	HCT-15 (IC_50_ = 1.43 ± 0.26 μM)	Colorectal cancer	[103]
β-carboline-3-carboxylic acid (6u)		β-carboline derivative	Bcl-2↓ Cyt-C↑	Induce apoptosis	A549 (IC_50_ = 5.61 ± 0.04 μM)	Lung cancer	[104]

↓, downregulate/inhibition; ↑, upregulate/activation

Fas/FasL is considered to be the most significant class of apoptosis-inducing molecules present in a variety of tumor cells [49]. A classic indole alkaloid, harmaline, as well as a β-carboline alkaloid, which was obtained from the seeds of *Peganum harmala*, has been examined for anti-gastric tumor activity both in vitro and in vivo. The preliminary results revealed that harmaline treatment resulted in the induction of extrinsic apoptosis, as evidenced by the upregulation of Fas/FasL expression and further activating caspase-8/-3 [56]. TNF-α, a powerful apoptogenic cytokine released by T cells or macrophages, binds to its homologous cell surface receptor, TNFR1, to trigger apoptosis via caspase cascades [4]. Scholarisines Q and R, which are both nor-monoterpenoid indole alkaloids, have been extracted from the fruits of *Alstonia scholaris* and exhibit strong cytotoxicity in glioma stem cells (GSCs). These two natural compounds have the ability to suppress proliferation and induce extrinsic apoptosis of GCSs by enhancing the TNF-α expression and caspase-3 cleavage, as well as impair the colony formation ability of GSCs [57]. The activation of the caspase cascade is the hallmark of apoptotic cell death, and caspase-8 is a vital marker of extrinsic apoptosis [58]. *N*-(*p*-coumaroyl) serotonin, an active indole alkaloid, was shown to induce apoptosis through the activation of caspase-8 and depolarization of mitochondrial membrane in glioblastoma cell lines [59].

### Targeting Bcl-2 family and Cyt-C

Bcl-2 family proteins are a class of proteins involved in the regulation of apoptotic signaling pathways, which control apoptosis by promoting or inhibiting mitochondrial dysfunction through the interaction of pro-apoptotic and anti-apoptotic proteins, and they are the most intensively studied class of proteins in apoptosis [52]. Bcl-2 protein has a strong inhibitory effect on apoptosis and exerts its anti-apoptotic activities mainly by regulating the integrity of mitochondrial and endoplasmic reticulum membranes and the release of Cyt-C [60]. Bax is a pro-apoptotic protein that has a direct antagonistic effect on Bcl-2. Tumorigenesis is commonly associated with abnormal expression of Bcl-2 family members. In multiple malignant tumors, Bim and Puma are highly methylated, and the expression level is reduced, thereby inhibiting the apoptosis of tumor cells [61, 62]. In addition, overexpression of Bcl-2 family members is closely associated with the resistance of tumor cells to chemotherapeutic agents. It has been shown that Mcl-1 upregulation can render several tumor cells resistant to the Bcl-2/Bcl-xL inhibitors ABT-737 and ABT-263 [63]. Cytochrome C (Cyt-C) was the first pro-apoptotic factor identified to be linked to mitochondria. Stimulated by the mitochondrial membrane potential, Cyt-C is released into the cytoplasm and then binds to Apaf-1, which activates procaspase-9 and triggers a caspase cascade leading to apoptotic cell death [64]. Small molecule compounds of natural origin have been widely used in the treatment of various diseases due to their strong anticancer activity and relatively low side effects. It was found that indole alkaloids have great potential to induce apoptosis by modulating the biological functions of Bcl-2 family-related proteins to exert anticancer effects. Here, we review some anticancer indole alkaloids and their derivatives that trigger apoptosis via targeting the Bcl-2 family proteins and Cyt-C (Table 1).

The β-carboline alkaloids possess a wide variety of pharmacological activities, and several studies have shown that β-carboline alkaloids could exert antitumor activities by inducing apoptosis. Harmine, as a natural β-carboline alkaloid derived from the seeds of *Peganum harmala*, has displayed an outstanding anti-angiogenesis effect, as well as an ideal inhibitory effect on the growth of various cancer cells. Harmine has shown to offer anti-gastric cancer effects, both in vitro and in vivo, which suppresses the proliferation, migration, and invasion and stimulates apoptosis of BGC-823 and SGC-7901 cells through the decreased level of cyclooxygenase-2 (COX-2), Bcl-2, and matrix metalloproteinase (MMP)-2 as well as increased Bax level [65]. Additionally, Ding et al. discovered that harmine could inhibit the proliferation and migration of breast cancer cells, as well as induce apoptosis via the downregulation of PDZ-binding motif (TAZ) and Bcl-2 protein and the upregulation of Bax protein in MDA-MB-231 and MCF-7 cells [66]. JKA97, as a novel analog of harmine, was also shown to possess strong antitumor effects both in vitro and in vivo. Thus, Luo et al. have demonstrated that JKA97 could trigger colon cancer cell apoptosis via a Bax-dependent and p53-independent manner [67]. Another research report that JKA97 could induce apoptosis and suppress the growth of breast cancer cells by upregulating p21 protein expression, regardless of p53 status [68]. The overexpression of p21 can lead to an induction of Bax and elicits apoptosis. These studies provide application value for the treatment of p53 mutated cancers.

*Nauclea subdita* bark is full of indole alkaloids with potential cytotoxic. Among them, subditine with the most potent anticancer activity exerts an anti-proliferation effect strongly and triggers apoptosis in LNCaP and PC-3 cells through the disruption of mitochondrial membrane potential, the release of Cyt-C, and the decrease in Bcl-2 and Bcl-xL levels, as well as accompanied with an increase in p53 in LNCaP cells [69]. Work by Hou et al. on the extraction of *Picrasma quassioides*, dehydrocrenatidine (DEC), a β-carboline alkaloid, exhibits outstanding growth inhibitory activity against hepatocellular carcinoma (HCC) cells both in vitro and in vivo through inducing apoptosis, as evidenced by the decreases in mitochondrial membrane potential, the mitochondrial dysfunction and the changes in apoptosis-related proteins, like Bax and Bcl-2 [70]. Caspase cascades play a central role in intrinsic and extrinsic apoptosis pathways. A study by Jeong et al. shows that a natural β-carboline alkaloid, 9-hydroxycanthin-6-one, could trigger human ovarian cancer cells’ apoptosis by activating caspase- and reactive oxygen species (ROS)-dependent pathways [71].

Further, Cyt-C induces cell death in caspase-dependent apoptosis, while it could be accumulated in the nucleus, which was related to the caspase-independent apoptosis [72]. For instance, in this work, the mechanism of a natural alkaloid, evodiamine-induced apoptosis in lung cancer cell A549, was explored in detail by Mohan et al. It was found that evodiamine-induced apoptosis by enhancing the release of Cyt-C from mitochondria and subsequently activating intrinsic and extrinsic apoptosis pathways, respectively. Among them, intrinsic apoptosis was induced by activating p53 phosphorylation and stimulating caspase-3 and caspase-9 cascades; however, DR5 and caspase-8 extrinsic apoptosis was mediated by the activation of nuclear Cyt-C [73]. Mcl-1 is an anti-apoptotic protein regulated by ubiquitin-mediated proteasomal degradation, which is essential for the survival of normal cells. It is overexpressed in various cancers, such as lung cancer, colon cancer, bladder cancer, etc., and is closely associated with poor prognosis [74, 75]. Therefore, targeting Mcl-1 is a promising therapeutic strategy for cancer. Evodiamine could significantly downregulate the expression of Mcl-1 protein to elicit apoptosis of 253J and T24 cells, and this effect is conducted by the inhibition of the mTOR/S6K1 pathway [76]. Besides, evodiamine combined with TRAIL would synergistically induce the apoptosis of bladder cancer cells [76], suggesting it could be used as an anticancer agent either alone or as an adjuvant in combination therapy.

Vinca alkaloids are a kind of important cancer drug which can prevent the division of cancer cells and lead to apoptosis. Vinorelbine, as a semisynthetic vinca alkaloid, is a chemotherapy drug commonly used to treat lung and breast cancer, as well as a microtubule-targeting agent. Vinorelbine has the competence to inhibit the migration and invasion of cancer cells, as well as induce G2/M arrest and cell apoptosis, which can be proved by the increase in Bax level and the decrease in Bcl-xL and Bcl-2 levels in H1975, HepG2, and HCT116 cells [77]. Another well-known alkaloid, vincamine, which is isolated from the leaves of *Vinca minor*, could be used as a dietary supplement and vasodilator in clinical. Besides, vincamine also displays anticancer activity against lung cancer cell line A549, with an IC_50_ value of 309.7 μM. Vincamine promotes the A549 cell death via the mitochondrial membrane potential change and the Cyt-C released, which profoundly triggers caspase-3-mediated apoptosis [78]. Vincamine may be a good candidate for clinical trials of anticancer drugs.

Gramine, as a simple indole alkaloid, possesses multiple bioactive properties, like anti-inflammatory, antiviral, and anti-angiogenesis effects [79–81]. Gramine was proved to inhibit angiogenesis by blocking transforming growth factor-β (TGF-β) signals and activating apoptotic genes to trigger cell death in oral squamous cell carcinoma (OSCC). The induction of apoptosis was mediated by enhancing the expression of Bax, Cyt-C, Apaf-1, caspase-9/3, and PARP [82]. More recently, the group adopted a pharmacophore fusion strategy to introduce nitrogen-containing heterocyclic pharmacophores and the terminal alkyne into gramine to enhance the ability to inhibit the gastric cancer cell MGC803 proliferation [83]. The results show that compound 16 h-treated could activate mitochondria-associated apoptotic pathway to induce apoptosis and suppress the proliferation of MGC803 cells in a dose-dependent manner, which can be proved by the increase in Bax and cleaved caspase-3/7/9 as well as the decrease in Bcl-2 [83]. Another simple indole alkaloid, indole-3-carbinol (I3C), a naturally occurring compound derived from multiple cruciferous vegetables, including cabbage, cauliflower, and radishes, contributes to inducing cancer cell growth arrest and exerts an anti-proliferative effect [84]. The previous experiment has shown that I3C-treated can induce increased expression of ROS in lung cancer H1299 cells, thereby activating the apoptosis-related signaling cascades, accompanied by the increased expression of pro-apoptotic proteins forkhead box O 3 (FOXO3), Bax, and Bim and the decreased expression of anti-apoptotic proteins Bcl-2 and Bcl-xL [84].

Bis-indole alkaloids have attracted widespread attention from researchers for their antitumor activity due to their specific structure, strong drug-like properties, high selectivity, and low toxicity, which are an important group of indole alkaloids and are available in large quantities. Cathachunine, a novel bis-indole alkaloid, which is derived from *Catharanthus roseus*, displayed a great antitumor effect on human leukemia cells and was shown to induce an intrinsic apoptotic pathway, as evidenced by the dysregulation of the Bcl-2/Bax ratio, the decrease in MMP, the release of Cyt-C, and the cleavage of caspase-3 and PARP [85]. This bis-indole alkaloid, (3′R)-hydroxytaberanelegantine C, was isolated from the methanol extract of *T. elegans* roots and exhibited potent apoptosis induction activity, accompanied by the level of Bcl-2 and XIAP decreased in HCT116, SW620, and HepG2 cells [86]. Furthermore, cimicifoetones is a dimeric indole alkaloid derived from the rhizomes of *Cimicifuga foetida,* which could enhance the expression of caspase-3/-8/-9, promote the cleavage of PARP, downregulate the anti-apoptotic proteins, Bcl-2, Bcl-xL, and upregulate the pro-apoptotic protein Bax, indicating a simultaneous activation of both extrinsic and intrinsic apoptotic pathways in HL-60 cells [87]. Malsy et al. found that staurosporine could induce apoptosis of pancreatic cancer cells through an intrinsic pathway, and the expression of anti-apoptotic factors Bcl-2 and Bad were reduced in PaTu 8988T cells, whereas staurosporine failed to induce apoptosis in colon cancer cells [88].

Marine bis-indole alkaloids are a large and growing class of secondary metabolites. Like this, 2,2-bis(6-Bromo-3-indolyl) ethylamine, a marine bis-indole alkaloid, which is isolated from *Didemnum candidum* and the New Caledonian sponge *Orina,* has been reported to trigger U937 human myelomonocytic lymphoma cells apoptotic cell death through the regulation of Bcl-2 family proteins upstream of caspase activation [89]. Likewise, another natural product, eusynstyelamide B is a bis-indole alkaloid derived from marine invertebrates, with half maximal inhibitory concentration (IC_50_) value of 5 μM in MDA-MB-231 cells, which can trigger apoptotic cell death but the detailed mechanism of action needs to be further explored [90].

Chetomin is a fungal metabolite derived from *Chaetomium cochliodes*, which is shown to trigger apoptosis in triple-negative breast cancer cell lines via the induction of calcium overload. An increase in intracellular Ca^2+^ concentration leads to the release of Cyt-C, which induces caspase-3-mediated cell death. In addition, the PI3K/mTOR pathway is also involved in chetomin-induced apoptotic cell death [91]. Chaetoglobosin A (ChA), a fungal extract produced by *Penicillium aquamarinium*, could effectively prevent the activation of chronic lymphocytic leukemia (CLL) cells and thus sensitize them to the PI3K and Bruton's tyrosine kinase (BTK) inhibitors treatment. Besides, ChA exhibits preferential induction of apoptosis in CLL cells, which was observed by the activation of caspase-3 and Annexin V-PE positive cells [92]. In addition, Duan et al. isolated 12 active alkaloids [11]-chaetoglobosin (1–12) from the endophytic fungus *Pseudeurotium bakeri* P1–1–1. By further analysis, [11]-chaetoglobosin B was found to have strong cytotoxic activity against MCF-7 cells and could activate apoptotic cell death via upregulating the expression of Bax, Cyt-C, and caspase-3 and downregulating the expression of Bcl-2 [93]. Yao et al. found that chaetominine, a secondary metabolite isolated from *Aspergillus fumigatus*, has the competence to reverse drug resistance [94]. In the K562 human leukemia cell line, resistance to adriamycin would be induced. And when treated with chaetominine, the resistance would attenuate and increase the sensitivity of K562/Adr to adriamycin. In addition, chaetominine induces apoptosis in K562/Adr cells by activating ROS production and modulating Bcl-2 family proteins expression [94].

Furthermore, reserpine is a well-known indole alkaloid extracted from *Rauwolfia serpentina*, which has sedative and antihypertensive effects [95]. Ramu et al. investigated the therapeutic effect of reserpine against DMBA-induced hamster buccal pouch carcinogenesis (HBP) model and reported that reserpine could inhibit DNA repair, cell proliferation, and invasion, as well as induce apoptosis through the suppression of TGF-β signals, which is accompanied with a decrease in ERCC1, Ku70, DNA-PKcs, cyclin D1, IL-6, and Mcl-1 and an increase in Bax, Cyt-C, Apaf-1, and PARP proteins expression [96]. Brucine, as an active ingredient of many traditional medicines, is isolated from the dried seed of *Strychnos nux vomica* L., which possesses the properties of antitumor, anti-angiogenic, and anti-proliferation effect [97]. In the hepatic tumor, brucine treatment could be observed that the expression of Cyclin D1 and Bcl-2 is decreased, whereas the expression of Bax and caspase-3 is enhanced, indicating that the anticancer activity of brucine is associated with the promotion of apoptosis [98]. Moreover, jerantinine B is a novel Aspidosperma indole alkaloid extracted from *Tabernaemontana corymbosa* leaf, and it could induce apoptosis in a time- and dose-dependent manner, as evidenced by downregulating the expression of Bcl-2 and Mcl-1 proteins, as well as activating the PARP and caspase-3/7 [99].

Isorhynchophylline (Rhy) is a classic indole alkaloid with various biological activities in *Uncaria rhynchophylla*, which can be used to treat diabetes, asthma, and cardiovascular diseases, but its anticancer activity and mechanism are less reported. Lee et al. found that Rhy has pronounced cytotoxicity against hepatocellular carcinoma HepG2 cells, and it can induce apoptosis and inhibit metastasis of tumor cells by regulating various signaling pathways [100]. Rhy was shown to inhibit the phosphorylation of p38, extracellular signal-regulated kinase (ERK), c-Jun *N*-terminal kinase (JNK), cAMP-response element-binding protein (CREB), c-Jun, Akt, and signal transducer and activator of transcription 3 (STAT3) and increase the phosphorylation of p53 in HepG2 cells. Besides, Rhy could downregulate the expression of Bcl-2, Bcl-xL, and surviving in a time-dependent manner to induce apoptosis [100]. Wang et al. investigated the cytotoxicity of bufothionine against gastric cancer (GC) cell lines and reported that bufothionine could inhibit the growth and evoked apoptotic cell death of GC cells by promoting the cleavage of caspase-3/8/9, downregulating the Bcl-2 level, and upregulating the Bax level [101]. Furthermore, bufothionine could also suppress PIM3 expression to exert anticancer activity, implying that PIM3 could be a potential therapeutic target for GC treatment [101]. Natural product-oriented synthetic derivatives are excellent sources of new drug candidates, especially in the field of anticancer therapy. New indole alkaloid derivatives are constantly being developed, and these structures readily bind to various receptors; thus, their synthesis represents a new prospect for lead compounds. CAA45 is a derivative of calothrixin A (CAA) with potent anticancer activities at nanomolar concentration. CAA45 was shown to elicit A549 cells apoptosis by promoting the release of Cyt-C, enhancing the expression of Bax and Bad, and downregulating the Bcl-2 expression. Additionally, the inhibition of Akt, activation of JNK, and upregulation of p53 protein observed in A549 cells may be associated with CAA45-induced apoptosis and autophagy, ultimately exerting an anti-proliferation effect on A549 cells [102].

As is well known, β-carboline alkaloids were naturally derived from the seeds of *Peganum harmala, Passiflora incarnata, and Banisteriopsis caapi,* with a wide range of biological properties, such as anxiolytic, antifungal, antiviral, antimicrobial, and anticancer activities [33]. And 2-oxindole is a representative active skeleton in drugs. A molecular hybridization strategy was used to link two pharmacophore groups to a new hybrid to obtain structurally diverse compounds [103]. Therefore, a series of β-carboline-linked oxindole hybrids have been synthesized, among them (*E*)-1-benzyl-5-bromo-3-{[1-(2,5-dimethoxyphenyl)-9*H*-pyrido[3,4-b]indol-3-yl]methylene}indolin-2-one (10 s) was identified as the most active hybrid with an IC_50_ value of 1.43 ± 0.26 μM in HCT-15 cells and it could effectively induce apoptosis through the enhanced expression of Bax and caspase-3/8/9, as well as the reduced expression of Bcl-2 [103]. Another bivalent β-carboline derivative, β-carboline-3-carboxylic acid (6u), was shown the best anticancer activity with an IC_50_ value of 5.61 μM against A549 cells, and this result indicated that the dimerization was a useful modification for antitumor effect enhanced. Furthermore, treatment with compound 6u would improve Cyt-C level and downregulate Bcl-2 expression, which in turn triggers apoptotic cell death [104].

### Targeting p53

The p53 is regarded as a prominent tumor suppressor protein that plays a key role in inducing cell cycle arrest, apoptosis, accelerating cell aging, and DNA repair in response to various genotoxic stresses [105]. It is also one of the most commonly mutated or silenced genes in cancer, with p53 mutations present in approximately 50% of human cancers, which cause loss of its normal function and ultimately lead to tumor growth. In response to DNA damage, p53 is activated and most probably binds directly to Bcl-2 and Bcl-xL, preventing their mitochondrial translocation by disrupting mitochondrial membrane integrity and then releasing Cyt-C to induce cell apoptosis [106–108]. Thus, targeting p53 protein as an anticancer therapy has attracted significant attention. Many indole alkaloids have been proven to generate cytotoxicity and induce apoptosis via the regulation of the balance of p53 protein in various cancer cells (Table 2).

**Table 2:** Indole alkaloids targeting p53, NF-κB, PI3K/Akt/mTOR, MAPKs, and other apoptotic pathways in cancer

Compound	Structure	Source	Target	Mechanism in RCD	Cancer cell line	Tumor type	Refs.
Flavopereirine		*Geissospermum vellosii*	p53, p21↑	Induce apoptosis	SW480 (IC_50_ = 15.33 μM); SW620 (IC_50_ = 10.52 μM); DLD1 (IC_50_ = 10.76 μM); HCT116 (IC_50_ = 8.15 μM); HT29 (IC_50_ = 9.58 μM)	Colorectal cancer	[111]
Chaetoglobosin K		*Diplodia macrospora*	p53↑	Induce apoptosis	OVCAR-3 (IC_50_ = 2.2 μM); A2780/CP70 (IC_50_ = 1.7 μM); IOSE-364 (IC_50_ = 11.1 μM)	Ovarian cancer	[112]
Khasuanine A		*Melodinuskhasianus*	p53↑ Bcl-2↓	Induce apoptosis	PC3 (IC_50_ = 0.74 ± 0.03 μM)	Renal carcinoma	[113]
(*Z*)-6-Chloro-3-(6,-dimethoxy-1,4-dihydroindeno[1,2-c]pyrazol-3yl))methylene) indolin-2-one		-	p53, p21, Bax↑	Induce apoptosis	HeLa (IC_50_ = 1.33 μM); A549 (IC_50_ = 2.44 μM); HEK-293 (IC_50_ = 2.97 μM); MDA-MB-231 (IC_50_ = 2.08 μM)	Cervical cancer; lung cancer; breast cancer	[114]
Melosuavine I		*Melodinus suaveolens*	p53↑ Bcl-2↓	Induce apoptosis	BT549 (IC_50_ = 0.89 ± 0.03 μM)	Breast cancer	[115]
Evodiamine		*Evodia rutaecarpa*	p53, Bax/Bcl-2↑	Induce apoptosis	HCT-116	Colorectal cancer	[116]
			p53, Bax↑ Bcl-2, NOD1↓	Induce apoptosis	HepG2, SMMC-7721 (IC_50_ ≈ 1 μM)	Hepatocellular cancer	[117]
Cryptolepine		*Cryptolepis sanguinolenta*	p53↑	Induce apoptosis	SCC-13; A431 (IC_50_ = 2.5–5.0 μM)	Non-melanoma skin cancer	[118]
Fumigaclavine C		*Aspergillus fumigatus*	NF-κB↓ Bax, Bad↑ Bcl-2, Bcl-xL↓	Induce apoptosis	MCF-7 (IC_50_ = 40–50 μM)	Breast cancer	[121]
Vinblastine		*Catharanthus roseus*	NF-κB↓	Induce apoptosis	NB4 cell	Human acute promyelocytic leukemia	[124]
Hapalindole H		*Fischerella muscicola*	NF-ĸB↓	Induce apoptosis	PC-3 (EC_50_ = 20 nM)	Prostate cancer	[125]
11-Methoxytabersonine		*Melodinus cochinchinensis*	PI3K/Akt/mTOR↓	Induce apoptosis	MOLT-4 (IC_50_ = 0.71 ± 0.08 μM); HL-60 (IC_50_ = 1.10 ± 0.07 μM)	T cell acute lymphoblastic leukemia	[128]
Staurosporine		*Streptomyces staurosporeus*	PI3K/Akt↓	Induce apoptosis	HepG2	Hepatocellular carcinoma	[129]
Brucine		*Strychnos nux vomica L*	PI3K/Akt/mTOR↓	Induce apoptosis	ME-180 (IC_50_ = 10–20 μM)	Cervical cancer	[130]
Meisoindigo		Indirubin derivative	PI3K/Akt↓	Induce apoptosis	U87 (IC_50_ = 20 μM)	Glioblastoma	[132]
1-(4-Methoxystyryl)-2-benzyl-9-(3-phenylpropyl)-β-carbolinium bromide		Harmine derivative	PI3K/Akt↓	Induce apoptosis	BGC-823 (IC_50_ = 0.46 μM); A375 (IC_50_ = 0.68 μM); KB (IC_50_ = 0.93 μM)	Colorectal cancer; gastric cancer; malignant melanoma	[133]
Harmalacidine		*Peganum harmala*	PTKs-Ras/Raf/ERK↓	Induce apoptosis	U937 (IC_50_ = 3.1 ± 0.2 μM)	Leukemia	[136]
Dehydrocrenatidine		*Picrasma quassioides*	ERK1/2, JNK1/2↑	Induce apoptosis	SAS, SCC-9	Oral squamous cell carcinoma	[137]
			ERK1/2↑ JNK1/2↓	Induce apoptosis	NPC-039, NPC-BM, RPMI-2650	Nasopharyngeal carcinoma	[138]
3α-Acetonyltabersonine		*Melodinus suaveolens*	MAPK↑	Induce apoptosis	U87 (IC_50_ = 1.7 μM); T98G (IC_50_ = 4.3 μM)	Glioblastoma	[140]
Flavopereirine		*Geissospermum vellosii*	ERK, p38 MAPK↑ AKT↓	Induce apoptosis	MDA-MB-231 (IC_50_ = 5.96 μM); MCF-7 (IC_50_ = 12.43 μM)	Breast cancer	[141]
Jerantinine B		*Tabernaemontana corymbosa*	c-Jun/JNK↑	Induce apoptosis	MV4–11 (IC_50_ = 0.3 μM); HL-60 (IC_50_ = 0.4 μM); KG1a (IC_50_ = 0.8 μM)	Acute myeloid leukemia	[142]
Sclerotiamides C		*Aspergillus sclerotiorum*	JNK, ERK, and p38↑	Induce apoptosis	HeLa (IC_50_ = 1.7 ± 0.1 μM)	Cervical cancer	[143]
L20		Calothrixin B derivative	p38 MAPK↑	Induce apoptosis	HEL (IC_50_ = 1.10 ± 0.05 μM); K562 (IC_50_ = 5.46 ± 3.09 μM); KG-1a (IC_50_ = 1.82 ± 1.08 μM)	Erythroleukemia	[144]
Brucine and strychnine		*Strychnos nux vomica* L	Wnt/β-catenin↓	Induce apoptosis	DLD1, SW480, and Lovo	Colon cancer	[146]
Nauclefine		*Nauclea subdita*	PDE3A-SLFN12↑	Induce apoptosis	HeLa (IC_50_ < 10 nM)	Cervical cancer	[149]
SOID-8		Spirooxindole derivative	JAK/STAT3↓	Induce apoptosis	A2058 (IC_50_ = 3.7 μM); A375 (IC_50_ = 5.3 μM)	Melanoma	[150]
3,10-Dibromofascaplysin		*Fascaplysinopsis reticulata*	E2F1↑	Induce apoptosis	K562 (IC_50_ = 318.2 nM); THP-1 (IC_50_ = 329.6 nM); MV4;11 (IC_50_ = 233.8 nM); U937 (IC_50_ = 318.1 nM);	Myeloid Leukemia	[152]
3-Chloro-5‴-fluorofradcarbazole A		Staurosporine derivative	FLT-3, c-kit↓	Induce apoptosis	MV4-11 (IC_50_ = 0.32 ± 0.03 μM)	Acute myeloid leukemia	[155]
Dregamine 5-bromo-pyridin-2-ylhydrazone		Monoterpene indole alkaloid derivative	BRCA1-BARD1↓ PUMA, PARP↑	Induce apoptosis	SKOV-3 (IC_50_ = 8.9 ± 2.5 μM); MDA-MB-468 (IC_50_ = 4.3 ± 1.2 μM); IGROV-1 (IC_50_ = 4.6 ± 1.5 μM)	Triple-negative breast cancer; ovarian cancer	[158]

↓, downregulate/inhibition; ↑, upregulate/activation

p21 is a downstream target protein of p53, and activation of p53 regulates the expression of p21 protein, induces cell cycle arrest, and interferes with DNA replication [109, 110]. In a study designed to assess the inhibitory effect of a natural alkaloid, flavopereirine, obtained from the *Geissospermum vellosii *in vitro and in vivo, performed by Li et al., flavopereirine-mediated growth suppression was attributed to activating p53 and p21 proteins expression, profoundly inducing the apoptotic cell death in colorectal cancer (CRC) cells [111]. Additionally, Li et al. found that chaetoglobosin K (ChK), obtained from fungus *Diplodia macrospora,* exhibits a powerful growth inhibition effect on platinum-resistant ovarian cancer cells. The experiment data indicate that it could activate the extrinsic apoptosis pathway by enhancing the expression of p53 protein and upregulating caspase-8, as well as block the cell cycle at the G2 phase [112]. Khasuanine A, a novel active ingredient extracted from the roots of *Melodinus khasianus* that has been further analyzed for its anticancer effects, was reported to induce apoptosis of PC3 cells through activating p53 protein and inhibiting Bcl-2 protein [113]. And Khan et al. has been synthesized a series of 1,4-dihydroindeno-[1,2-c] pyrazole linked oxindole conjugates, among them (Z)-6-Chloro-3-(6-dimethoxy-1,4-dihydroindeno[1,2-c]pyrazol-3yl))methylene) indolin-2-one (12d) shows the excellent cytotoxicity with an IC_50_ value of 1.33 μM against HeLa cells. Besides, compound 12d could block the cell cycle at the G2/M phase and trigger apoptotic cell death by upregulating the p53, p21, and Bax proteins [114].

A further experiment by Fang et al. showed that melosuavine I, a novel bis-indole alkaloid derived from *Melodinus suaveolens*, possessed potent cytotoxicity with an IC_50_ value of 0.89 μM on BT549 cells. The preliminary screening revealed that melosuavine I treatment resulted in the induction of apoptosis by stimulating the p53 expression and inhibiting the Bcl-2 level, in addition to the activation of the anti-proliferation effect in breast cancer cells [115]. Moreover, Zhao et al. reports that evodiamine can increase the activity of p53 and the Bax/Bcl-2 ratio to induce apoptosis in HCT-116 cells. Besides, Janus kinase 2 (JAK2)/STAT3 pathway plays a vital role in the metastasis inhibition of HCT-116 cells [116]. Nucleotide-Binding Oligomerization Domain 1 (NOD1) shows high expression in a variety of tumor tissues and is closely associated with apoptosis. NOD1 can activate NF-κB-dependent as well as MAPK-dependent gene transcription. Evodiamine could block the cell cycle at the G2/M phase and suppress cell proliferation through the upregulation of p53 and Bax, as well as the downregulation of Bcl-2, CyclinB1, and cdc2 proteins. Besides, it triggers apoptosis remarkably in HepG2 and SMMC-7721 cells via suppressing the NOD1 signaling pathway in vitro and in vivo [117]. Cryptolepine, an active plant alkaloid, could induce DNA damage and then activate the p53 expression, induce the disruption of the Bcl-2/Bax ratio, which in turn leads to Cyt-C release from mitochondria, ultimately initiating the apoptosis in human non-melanoma skin cancer cells [118].

### Targeting NF-κB pathway

The Nuclear factor kappa-B (NF-κB) comprises a family of transcription factors involved in various biological responses, such as cell proliferation, survival, differentiation, and apoptosis. Generally, NF-κB exists in the cytoplasm in the form of p50 and p65 dimers and is bound to the specific inhibitor IκB in an inactive state. When subjected to certain stimuli, the IκB protein is degraded by phosphorylation, resulting in the activation and release of NF-κB dimers, which are transferred from cytoplasm to nucleus, combined with target genes, and promoted transcription [119]. However, in cancer cells, NF-κB exists in an active form, which can induce the expression of pro-survival genes, such as inhibitors of apoptosis (IAPs), which leads to the cell’s uncontrolled growth. Thus, blocking NF-κB activity can regulate the survival/death balance of tumor cells [120]. In addition, Bcl-2 has a site that can specifically bind to NF-κB, so inhibition of NF-κB activity can also inhibit Bcl-2 expression, which in turn promotes tumor cell apoptosis. Consequently, we have explored some typical active indole alkaloids derived from various natural sources, which stimulate apoptosis primarily through inhibiting the NF-κB signaling pathway (Table 2).

A study by Li et al. showed that marine-derived fungus *Aspergillus fumigatus* have potential anticancer effects on breast cancers and its active component, fumigaclavine C, induces apoptosis through upregulating the Bax and Bad levels as well as downregulating the Bcl-2 and Bcl-xL levels in a NF-κB-dependent manner. In addition, the induction of apoptosis might relate to the p53 family expression and the activation of the MAPK signaling pathway [121]. Vinblastine belongs to the vinca alkaloid family as an anti-microtubule agent, which has been used as a single clinical drug or in combination with other drugs to treat a variety of cancers, including leukemia, breast cancer, lung cancer, and other solid tumors. Nowadays, vinblastine is reported to trigger apoptosis in different tumor cells via multiple signaling pathways [122, 123]. In a study, the mechanism of anticancer effect of vinblastine on human acute promyelocytic leukemia NB4 cells was reported, and it was found that vinblastine could induce an apoptotic response by downregulating NF-κB expression, sustaining JNK activation, and finally triggering caspase cascade. In addition, it will also cause changes in p53 and DNA fragmentation [124]. Moreover, hapalindole H (Hap H), extracted from *Fischerella muscicola* with a median effective concentration (EC_50_) value of 20 nM, induced toxicity in the PC-3 prostate cancer cells, and it showed potential NF-ĸB p65 inhibitory activity on PC-3 cells, and this inhibition would lead to apoptotic cell death. Besides, the induction of apoptosis was also examined by affecting the mitochondrial transmembrane potential [125].

### Targeting PI3K/Akt/mTOR pathway

The PI3K/Akt/mTOR signaling pathway plays an important role in the physiological activities of tumor cells, such as energy metabolism, cell proliferation, invasion, apoptosis, and cell cycle. The PI3K/Akt/mTOR signaling pathway is aberrantly activated in many human malignant tumors, and blocking the PI3K/Akt/mTOR pathway has emerged as a new therapeutic target for multiple cancer cells [126]. This pathway is closely related to other apoptotic pathways as well suppressed by the negative regulator phosphatase and tensin homologue deleted on chromosome ten (PTEN) [127]. Herein, several active indole alkaloids obtained from natural sources, such as 11-methoxytabersonine, staurosporine, and brucine, exert their anticancer activity and trigger apoptosis by mainly suppressing the PI3K/Akt/mTOR pathway (Table 2).

In one study, an aspidospermine alkaloid, 11-methoxytabersonine (11-MT), obtained from a folk medicine *Melodinus cochinchinensis* exhibits potent cytotoxicity on human T cell acute lymphoblastic leukemia (T-ALL) cells. 11-MT can inhibit proliferation and elicit apoptosis through the accumulated ROS and the increased calcium concentration via downregulating the PI3K/Akt/mTOR pathway in MOLT-4 cells [128]. Staurosporine, a protein kinase inhibitor isolated from *Streptomyces staurosporeus*, has been proved to suppress the viability and induce apoptosis of HepG2 hepatocellular carcinoma cells, which was associated with the downregulation of PDK1 protein and Akt phosphorylation [129]. More recently, work has shown that brucine suppresses inflammation and cell proliferation and induces a PI3K/Akt/mTOR-mediated apoptotic pathway in the cervical cancer ME-180 cells, as evidenced by modulating inflammatory and apoptotic proteins expression, including TNF-α, NF-ĸB, COX-2, IL-6, Cyclin D1, Bcl-2, Bax, etc. [130]. Meisoindigo is an indirubin derivative with the highest antitumor activity, which has been used to treat chronic myeloid leukemia (CML) via the induction of cell cycle arrest at the G0/G1 phase, thus suspending the growth of leukemic cells [131]. Moreover, it was reported that meisoindigo could inhibit proliferation and trigger apoptotic cell death of glioblastoma U87 cells by blocking the PI3K/Akt pathway and suppressing the NF-κB p65 from the nucleus to the cytoplasm [132]. Additionally, 1-(4-methoxystyryl)-2-benzyl-9-(3-phenylpropyl)-β-carbolinium bromide (3c) is a newly synthesized harmine derivative. The group investigated the antitumor effects of compound 3c and found that it could induce CRC cell apoptosis by inhibiting the PI3K/Akt signaling pathway and promoting the accumulation of ROS. Besides, in in vivo experiments, compound 3c treatment would suppress tumor growth and attenuate tumor weight [133].

### Targeting MAPK pathway

MAPK signaling pathway regulates various biological processes through different cellular mechanisms, including cell proliferation, differentiation, and apoptosis. MAPK could be classified into three distinct cascades: ERK1/2, JNK1/2, and p38 MAPK [134]. Abnormal and overexpression of MAPK in tumors may contribute to the development of cancer cells, leading to uncontrolled proliferation and resistance to apoptosis. Therefore, we agree that the MAPK signaling pathway is an important target for anticancer drugs, and an effective blockade of its activation may be a promising therapeutic approach for the development of novel anticancer drugs (Table 2).

The ERK1/2 pathway is a signal axis consisting of Ras-Raf-MEK-ERK1/2. Ras is stimulated by some cytokines and binds to Raf, which is then transferred from the cytoplasm to the cell membrane. MEK binds to activated Raf at the cell membrane, activates ERK1/2 after phosphorylation, and subsequently regulates the expression of various apoptotic factors [135]. A study showed that the toxic plant *Peganum harmala* is rich in various indole alkaloids, among which harmalacidine has a strong inhibitory effect on U937 cells with an IC_50_ value of 3.1 ± 0.2 μM. The group also studied the mechanism of action of harmalacidine and found that it can elicit apoptosis in leukemic cells, which is regulated by the inactivation of the Ras/Raf/ERK pathway [136]. Dehydrocrenatidine (DC), a β-carboline alkaloid derived from *Picrasma quassioides*, has been proved to exhibit analgesic effects but its anticancer activity is not yet known. According to Ho et al., DC could block the cell cycle at sub-G1 and G2/M phases as well as trigger apoptotic cell death of SAS and SCC-9 cells by upregulating MAPKs proteins ERK1/2 and JNK1/2 expression levels, thus regulating apoptosis-related proteins, like Bax, Bcl-2, Bcl-xL, and Bak [137]. In addition, this group also investigated the effects of DC on nasopharyngeal carcinoma (NPC). DC could also induce apoptosis by enhancing phosphorylation of ERK1/2 and inhibiting phosphorylation of JNK1/2 in human NPC cells [138].

Cancer stem cells are small groups of cells in cancers, which play an important role in tumor growth, and metastasis, and therefore, targeting cancer stem cells may have a better therapeutic effect [139]. A cytotoxic indole alkaloid, 3α-acetonyltabersonine, was found to exhibit better cytotoxicity and anti-proliferation effect on glioblastoma stem cells than on glioblastoma cell lines. Moreover, 3α-acetonyltabersonine was shown to inhibit the DNA damage repair process and induce apoptosis through the activation of MAPK phosphorylation (p-ERK and p-JNK). And the in vivo experiment showed that 3α-acetonyltabersonine could prolong the survival time of the glioblastoma mouse model [140]. Additionally, a β-carboline alkaloid, flavopereirine, was also found to trigger cell cycle arrest, suppress the activation of AKT, and increase the ERK and p38 MAPK expression level, ultimately resulting in the induction of apoptosis in MDA-MB-231 cells [141]. According to related literature reports, a natural indole alkaloid, Jerantinine B, has an effective targeting effect on acute myelocytic leukemia (AML) cells and promotes ROS-induced activation of the c-Jun/JNK pathway, which also contributes to the occurrence of apoptosis. Besides, the upregulation of apoptotic markers caspase-3 and cleaved PARP could also prove to apoptosis of cancer cells [142].

In a study, sclerotiamides C, derived from *Aspergillus sclerotiorum,* is a notoamide-type alkaloid with a novel 2,2-diaminopropane framework, which was shown to arrest the cell cycle and induce apoptosis in HeLa cells through enhancing the phosphorylation of JNK, ERK, and p38, as well as stimulating the activation of apoptosis-related proteins, such as Cyt-C, Bax, and p53, indicating that the MAPK pathway plays a vital role in modulating cell proliferation and apoptosis in HeLa cells [143]. Calothrixin B is a natural alkaloid compound with a unique indolo[3,2-j] phenanthridine structure that has been reported to exhibit low cytotoxicity in leukemia cells. While L20, as a novel derivative of calothrixin B, has strong anti-proliferation activity, it can induce mitochondria-mediated apoptosis as well as G2/M phase block, which can be carried out through DNA damage and targeting p38 MAPK pathway, also accompanied by the downregulation of p-ERK and c-Myc protein expression in the HEL cells [144]. Anyway, L20 has the potential as a novel chemotherapeutic agent in the treatment of erythrocytic leukemia.

### Targeting other apoptotic pathways

The Wnt/β-catenin signaling pathway is involved in the occurrence and development of various tumors. In tumor cells, the Wnt pathway is abnormally activated, and the degradation of β-catenin is inhibited. The free β-catenin can bind with T cell factors/lymphatic enhancer factors (TCF/LEF) to enter the nucleus, which promotes the transcription of downstream target genes, Cyclin D1 and c-Myc, thus affecting cell proliferation, apoptosis, and migration [145]. Ren et al. found that brucine and strychnine, which are isolated from the seeds of *Strychnos nux vomica* L., could induce apoptosis of colon cancer cells, as evidenced by the expression of DKK1 and APC increased, as well as the β-catenin, c-Myc, and p-LRP6 levels decreased [146]. Additionally, it was reported that some phosphodiesterase 3 (PDE3) enzyme inhibitors were found to be cytotoxic to cancer cells and combined with PDE3A to promote apoptosis independent of the canonical cyclic adenosine monophosphate (cAMP) and cyclic guanosine monophosphate (cGMP) hydrolysis activity of PDE3A [147, 148]. More recently, work has shown that nauclefine, a novel indole alkaloid natural product isolated from the bark of *Nauclea subdita*, triggers a PDE3A-SLFN12-dependent pathway to elicit apoptosis in the HeLa cells without affecting the PDE activity [149]. It suggests that nauclefine, a special PDE3A regulator, can induce cell death without affecting its typical functions, showing potent physiological activity. The JAK/STAT3 signaling pathway is abnormally activated in cancer cells and plays a key role in cell survival and apoptosis. SOID-8 as a spiro[pyrrolidin-3,39-oxindole] derivative exerts antitumor effects on melanoma cells. It is found that SOID-8 inhibits the phosphorylation of JAK2 and STAT3 to suppress growth and induce apoptosis in A2058 and A375 cells [150].

E2F1 as a major regulator of cell survival participates in the regulation of cell cycle progression and apoptosis [151]. 3,10-Dibromofascaplysin (DBF) (a halogenated fascaplysin alkaloid) derived from *Fascaplysinopsis reticulata*. The effect of DBF on leukemia cells was tested by Spirin et al., and the results showed that after DBF treatment of myeloid leukemia cells, the expression of E2F1 is upregulated, leading to the blockage of the S and G2 phases’ cell cycle, which in turn may lead to apoptosis [152]. FMS-like tyrosine kinase 3 (FLT-3) and c-kit are type III receptor tyrosine kinases that play an important role in cell survival, proliferation, and apoptosis. FLT-3 and c-kit are the most frequently mutated genes in AML cells, and the suppression of FLT-3 and c-kit can lead to apoptosis in AML cells [153, 154]. 3-chloro-5‴-fluorofradcarbazole A is a staurosporine derivative that was reported to downregulate the expression of FLT-3, CDK2, and c-kit and effectively triggered apoptosis in MV4-11 cells [155].

Mutations in BRCA1 and the BRCA1-associated ring domain protein (BARD1) make carriers more likely to develop breast and ovarian cancers [156]. The heterodimeric complex (BRCA1-BARD1) formed by BRCA1 and BARD1 acts as an E3 ubiquitin ligase that promotes DNA double-strand break (DSB) repair through homologous recombination (HR) [157], and inhibition of this complex is an effective therapeutic approach to inhibit homologous DNA repair (HDR), thereby reversing the onset of therapeutic resistance. Dregamine 5-bromo-pyridin-2-ylhydrazone (BBIT20) is a natural monoterpene indole alkaloid derivative as well as an HDR inhibitor [158]. By inhibiting the interaction between BRCA1-BARD1, BBIT20 triggered BRCA1 cellular relocation, cell cycle arrest, and downregulated the expression of the HDR-related proteins, possibly by proteolytic degradation after the destruction of the BRCA1-BARD1 complex, thus enhancing DNA damage, ROS generation and apoptosis in triple-negative breast cancer and ovarian cancer cells [158]. In addition, the induction of apoptosis by BBIT20 was associated with the expression of Puma, and cleaved PARP proteins increased (Table 2).

## Targeting autophagic signaling pathways with indole alkaloids in cancer

Autophagy is a highly conserved biological phenomenon, a process by which eukaryotic cells degrade their damaged organelles and proteins by lysosomes and produce amino acids, free fatty acids, and other substances to recycle energy so that cells can adapt to hypoxia and starvation [159–161]. Autophagy is induced in response to different stressors, such as lack of nutrients and growth factors, DNA damage, hypoxia, or energy deficiency, thus allowing the cell to compensate for the damage sustained [162]. The basic processes of autophagy include autophagy induction, autophagosome biogenesis, autolysosome formation, and degradation. In the case of normal physiology or nutritional insufficiency, autophagy acts more as a protective mechanism to maintain the normal physiological functions of cells. However, when a particular threshold is exceeded, excessive autophagy will promote cellular damage resulting in autophagic cell death, and this effect is being applied to cancer treatment [163]. During metabolic stress and cell damage, autophagy can act as a cell growth inhibitory mechanism to prevent stressed or damaged cells from becoming tumor cells, thus playing a role in tumor inhibition [164]. The role of autophagy in the development of cancer is rather complex, and it is a “double-edged sword” [165]. Whether the effect of autophagy on tumor growth is positive or negative depends upon the tumor tissue, the tumor type, and the stage of cancer development, as well as the active degree of the autophagic process. On the one hand, excessive activation of autophagy could induce autophagic cell death, thus suppressing tumor progression; on the other hand, autophagy inhibition may strengthen the action of chemotherapeutic agents by re-sensitizing resistant cancer cells [166, 167].

**Fig. 5:**
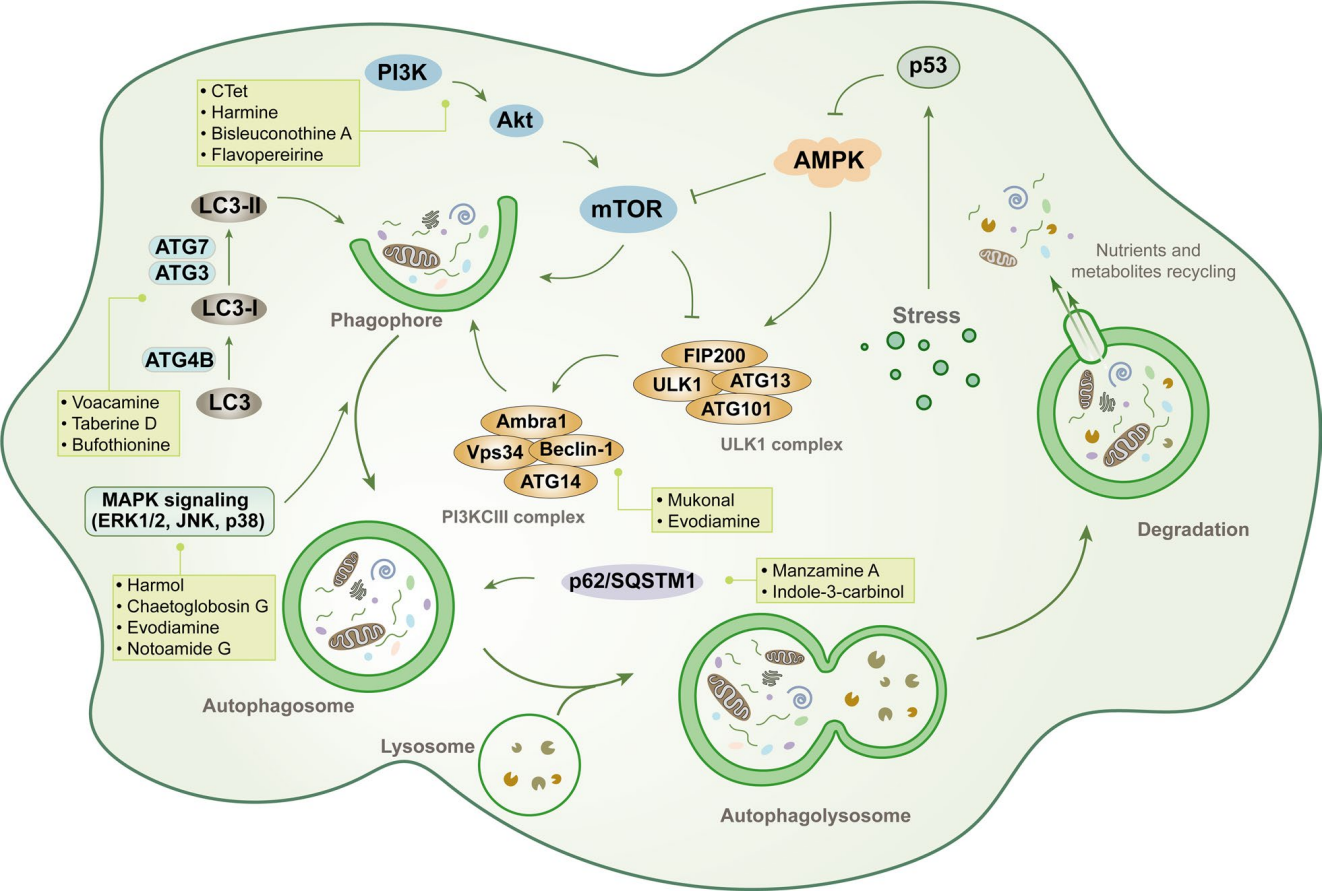
Schematic overview of autophagy. The autophagy process begins with the formation of phagophore structures. The phosphatidylinositol 3 kinase (PI3K)/protein kinase B (Akt) signaling pathway can activate the mammalian target of rapamycin (mTOR) and then regulate the initiation of autophagy through the unc-51-like kinase 1 (ULK1) complex. ULK functions in a complex with autophagy-related genes (ATG) 13, ATG101, and focal adhesion kinase interacting protein of 200 kD (FIP200), and the activation of ULK1 complex occurs through the stimulation of adenosine 5′-monophosphate-activated protein kinase (AMPK) and the suppressing of mTOR. AMPK negatively regulates mTOR, whereas cytoplasmic p53 can activate mTOR by inhibiting AMPK. Autophagy is also modulated by the PI3KCIII interactive complex, which consists of Beclin-1, vacuolar protein sorting 34 (Vps34), Ambra1, and ATG14. The expression of ATG4B, ATG3, and ATG7 converts light chain 3 (LC3) protein from its LC3-I form to LC3-II and promotes autophagosome formation. The combination of mature autophagosome and lysosome leads to autolysosome formation. Ultimately, autolysosomes are degraded by lysosomal hydrolases and recycling nutrients for use in the metabolism

In order to respond to external stimuli accurately, autophagy needs to be strictly regulated by a variety of autophagy-related genes (ATG) and proteins. Besides, autophagy is also modulated by a series of complex signaling pathways [168]. The unc-51-like kinase 1 (ULK1) complex [ULK1/ATG13/ATG101/focal adhesion kinase interacting protein of 200 kD (FIP200)], as a serine/threonine protein kinase, regulates the autophagy initiation function [169]. In addition, the ULK1 complex also acts as a sensor for upstream signaling pathways, thus exerting different effects on autophagy. Its upstream signaling pathways mainly include the mTOR, adenosine 5′-monophosphate-activated protein kinase (AMPK), and p53 pathways [170, 171]. mTOR is an extremely important mediator in the initiation of autophagy, which is regulated by nutrients and growth factors. AMPK is a negative regulator of mTOR, and activated AMPK prevents the activation of mTOR mainly through the phosphorylation of tuberous sclerosis complex 1/2 [172], thereby stimulating the early occurrence of autophagy. mTOR and AMPK inversely regulate the ULK1 complex through a sequence of phosphorylation events [173, 174]. Moreover, under high-nutrient conditions, mTOR is activated and phosphorylates ATG13 and ULK1 by binding to the ULK1 complex, thus inhibiting the activity of ULK1 kinase, disrupting the interaction of ULK1 with AMPK, and further inhibiting autophagy [175]. The p53 protein can control autophagy through transcriptional regulation. The induction of autophagy under certain conditions is not dependent on the mTOR pathway but induces the occurrence of autophagy through phosphatidylinositol 3 kinase complex III (PI3KCIII) binding to Beclin-1 [176]. ULK1 complex is closely related to Beclin-1, and ULK1 can phosphorylate ATG14, which promotes the binding of Beclin-1 to vacuolar protein sorting 34 (Vps34), ultimately participating in the regulation of autophagy [177]. Light chain 3 (LC3) is a homolog of the yeast ATG8 gene in mammalian cells, and it is also a protein that enables membrane structures to be aggregate and develop into phagocytic vesicles [178]. LC3 is first cleaved by ATG4B to form the water-soluble LC3-I and then binds with phosphatidylethanolamine to form lipid-soluble LC3-II through ATG7 and ATG3, which is localized on the membrane of intracellular autophagosome [179]. The amount of LC3-II is proportional to the number of autophagosomes, serving as a promising indicator of the degree of autophagosome formation (Fig. 5).

Accordingly, the potential role of autophagy in cancer is quite complex and has been linked to both the induction and suppression of neoplasia [180, 181]. Interestingly, in cancer treatment, diverse indole alkaloids derived from natural sources, such as harmol, chaetoglobosin G, bisleuconothine A, manzamine A, rhynchophylline, and voacamine, have been reported to exert therapeutic effects by targeting autophagy, and the main autophagy-related signaling pathways involve MAPKs pathway, PI3K/Akt/mTOR pathway, JAK2/STAT3 pathway, p62/SQSTM1, Beclin-1, ROS, etc. These signaling pathways are usually interrelated and can be integrated into the tumor regulatory network of autophagy-related proteins, which ultimately affect tumor cell survival [160, 182]. Therefore, it is necessary to gain an in-depth understanding of the biological relationship between autophagy and cancer, which is of great significance to exploring potential cancer therapeutic targets, and the discovery of more natural indole alkaloids will provide new opportunities for the development of novel anticancer drugs. In the following section, we have discussed the research progress of the interaction between indole alkaloids and related signaling pathways in cancer treatment (Table 3).

**Table 3:** Indole alkaloids targeting autophagic signaling pathways in cancer

Compound	Structure	Source	Target	Mechanism in RCD	Cancer cell line	Tumor type	Refs.
Harmol		*Peganum harmala*	ERK1/2↑	Induce autophagy	A549 cell	Non-small Cell Lung Cancer	[188]
Chaetoglobosin G		*Chaetomium globosum*	MEK, ERK↓ LC3-II↑	Induce autophagy	A549 (IC_50_ = 5–10 μM)	Lung cancer	[189]
Evodiamine		*Evodia rutaecarpa*	Calcium/JNK↑	Induce autophagy	U87-MG (IC_50_ = 5.21 μM)	Glioblastoma	[193]
Notoamide G		*Aspergillus ochraceus*	p38, JNK↑	Induce autophagy	HepG2 (IC_50_ = 1.31 ± 0.11 μM); Huh-7 (IC_50_ = 1.54 ± 0.08 μM);	Hepatocellular carcinoma	[194]
CTet		Indole-3-carbinol derivative	Akt↓ mTOR↓	Induce autophagy	MDA-MB-231 (IC_50_ = 1.00 ± 0.01 μM); MCF-7 (IC_50_ = 1.32 ± 0.03 μM)	Breast cancer	[200, 201]
Bisleuconothine A		*Leuconotis griffithii*	Akt/mTOR↓	Induce autophagy	A549 (IC_50_ = 8 μM)	Lung cancer	[203]
Harmol		*Peganum harmala*	Akt/mTOR↓	Induce autophagy	U251MG	Glioma	[202]
Harmine		*Peganum harmala*	PI3K/Akt/mTOR↓	Induce autophagy	B16 (IC_50_ = 44.92 μM)	Melanoma	[204]
Flavopereirine		*Geissospermum*	Akt/p38MAPK↓	Inhibit autophagy	MDA-MB-231	Triple-negative breast cancer	[205]
4-Bromo-*N*-(2-(dimethylamino)ethyl)-5,12-dihydroindolo[3,2-a]carbazole-6-carboxamide		Racemocin B derivative	p62, LC3-II↑	Inhibit autophagy	MDA-MB-231 (IC_50_ = 1.06 ± 0.10 μM); MCF-7 (IC_50_ = 2.00 ± 0.24 μM)	Breast cancer	[215]
Manzamine A		*Haliclona* sp., *Xestospongia* sp. and *Pellina* sp.	p62/SQSTM1, LC3-II↑	Inhibit autophagy	AsPC-1 and PANC-1	Pancreatic cancer	[216]
Indole-3-carbinol		Cruciferous vegetables	p62↓ LC3-II↑	Induce autophagy	EAC cell	Ehrlich ascites carcinoma	[219]
Mukonal		*Murraya koenigii*	Beclin-1↑	Induce autophagy	MDA-MB-231; SK-BR-3 (IC_50_ = 7.5 μM)	Breast cancer	[228]
Evodiamine		*Evodia rutaecarpa*	Beclin-1↑	Induce autophagy	SGC-7901	Gastric cancer	[229]
Bufothionine		Toad Bufo bufo gargarizans Cantor	JAK2/STAT3↓	Induce autophagy	SMMC-7721	Hepatocellular carcinoma	[233]
Reduced scytonemin		*Nostoc commune*	ROS↑ LC3-II↑	Induce autophagy	Jurkat (IC_50_ = 1.8 μM)	Leukemia	[239]
Fascaplysin		*Fascaplysinopsis* sp.	ROS, p8↑	Induce autophagy	HUVECs (EC_50_ = 1.3 μM)	Vascular endothelial cell	[241]
EE-84		Aplysinopsin derivative	ER stress↑ PERK-eIF2α-ATF4-CHOP↑	Induce autophagy	K562 (GI_50_ = 19.07 ± 0.80 μM)	Myeloid leukemia	[242]
Voacamine		*Peschiera fuchsiaefolia*	LC3-II/LC3-I↑	Induce autophagy	U-2 OS/WT and U-2 OS/DX	Osteosarcoma	[243]
Taberine D		*Tabernaemontana corymbosa*	LC3-II/LC3-I↑	Inhibit autophagy	HL-60, A549, SMMC-7721, MCF-7 (IC_50_ < 10 μM)	Leukemia; lung cancer; liver cancer; breast cancer	[244]

↓, downregulate/inhibition; ↑, upregulate/activation

### Targeting MAPK pathway

The MAPK signal transduction pathway is an essential regulatory mechanism in eukaryotic cells, and the main role of this pathway is to receive membrane receptors and convert and transmit relevant signals to the nucleus, which is participated in regulating cell growth and differentiation and can affect the occurrence of autophagy [183–185]. MAPK signaling pathway has three main family members: ERK, JNK, and p38 MAPK [134]. Among the ERK signaling pathways, ERK1 and ERK2 are the most studied pathways. Currently, more than 70 substrates are known for ERK, including nuclear transcription factors that regulate various cell behaviors [186]. The classical ERK pathway is mainly the Ras-Raf-MEK-ERK cascade, in which the guanosine triphosphate subtype of Ras protein binds to the serine/threonine kinases of Raf protein to activate the dual kinase action of Raf, which then sequentially activates MEK1/2 and ERK1/2 to regulate cell differentiation, proliferation, and autophagy [186, 187]. The activated ERK signaling pathway can not only activate autophagy by itself but also trigger autophagy by upregulating the expression of autophagy-related proteins such as LC3 and p62 [187]. Harmol, a β-carboline alkaloid, which is isolated from *Peganum harmala*, was shown to exert a cytotoxicity effect and trigger autophagic cell death in A549 NSCLC cells. And the harmol-induced autophagy was mediated through the activation of the ERK1/2 pathway. There was an increase in autophagosome formation, p-ERK1/2 levels, and LC3-II levels [188]. Furthermore, MEK/ERK signaling pathway is also an important downstream signaling pathway of EGFR. Chaetoglobosin G (CG), as a cytochalasin alkaloid derived from the fungal secondary metabolite, possesses anticancer activity. CG treatment stimulated autophagic cell death of A549 lung cancer cells and was associated with G2/M phase cell cycle arrest, inhibition of cyclinB1 protein, and upregulation of p21 protein. CG-induced autophagic effect was mediated primarily by downregulating the expression of p-EGFR, p-MEK, and p-ERK proteins and upregulating the expression of LC3-II protein, suggesting that CG could elicit autophagy via the EGFR/MEK/ERK signaling pathway [189].

Moreover, the JNK pathway has been related to autophagic-induced cell death. JNK can block the combination of Bcl-2 and Beclin-1 by phosphorylating Bcl-2 family proteins, which facilitates the binding of Beclin-1 to Vsp34 and the formation of PI3KIII complex, thereby positively regulating autophagy [190]. p38 MAPK is a class of tyrosine kinases with five different subtypes, including p38α, p38β1, p38β2, p38γ, and p38δ. p38α is a negative autophagy regulator, which can make the conversion of LC3-I to LC3-II fail through the phosphorylation of ATG5, and it can also downregulate ERK activity and significantly inhibit the activation of cell autophagy [191]. MAPK-activated protein kinase 2 (MK2), a member of the p38 signaling pathway, can also directly phosphorylate the S90 site of Beclin-1, which is essential for tumor suppression of Beclin-1 protein and can upregulate autophagy levels, maintain cellular homeostasis, and prevent tumorigenesis [192]. Liu et al. demonstrated that in U87-MG glioblastoma cells, evodiamine activates calcium release from the endoplasmic reticulum (ER) as well as stimulates the JNK signaling pathway, thereby resulting in the induction of autophagy. Besides, the calcium channel was also involved in mitochondrion-mediated apoptosis [193]. Another study by Hu et al. found that notoamide G, a novel alkaloid derived from the fungus *Aspergillus ochraceus,* possessed cytotoxic activities against HCC cell lines, which could inhibit the proliferation of HepG2 and Huh-7 cells through triggering autophagic cell death mediated by the activation of p38/JNK signaling pathway. Besides, it could be observed that the autophagy-related proteins, Beclin-1 and LC3-II, were upregulated and thus promoted the phosphorylation of p38 and JNK, leading to the induction of autophagy [194].

### Targeting PI3K/Akt/mTOR pathway

PI3K/Akt/mTOR pathway plays an important role in regulating the cell cycle, cell growth, and translation. Targeting PI3K/Akt/mTOR pathway not only elicits apoptosis to inhibit tumor cell proliferation but also triggers autophagy [195]. There are three isoforms of PI3K, among which class I PI3K, and class III PI3K are mainly involved in regulating autophagy. In general, the activation of class I PI3K tends to inhibit autophagy, and the class I PI3K/Akt/mTOR signaling pathway is a key component in the negative regulation of autophagy. Class I PI3K negatively regulates autophagy mainly by activating Akt, which phosphorylates TSC1/2, thus further activating mTOR [196]. In contrast, class III PI3K is the mammalian homolog of Vps34 in yeast, and phosphatidylinositol 3-phosphate (PI3P) is its only phosphorylation product. Class III PI3K is mainly involved in the formation of membrane and autophagosome in the early stage of autophagy and belongs to the positive regulators of autophagy [197]. mTOR is one of the important substrates of Akt, which can form two complexes, mTORC1, and mTORC2, in which the most important component of mTORC1 is Raptor, while the most important component of mTORC2 is Rictor. Under normal cell conditions, mTOR inhibits autophagy by phosphorylating mAtg13 and ULK1; in contrast, mTOR kinase is inhibited after rapamycin stimulation or nutrient starvation [198, 199].

Additionally, PI3K/Akt/mTOR signaling pathway is aberrantly activated in human melanoma, breast cancer, lung cancers, and other tumors. Targeting the PI3K/AKT/mTOR pathway provides a new therapeutic idea for treating these diseases [163, 195]. CTet, as an indole-3-carbinol cyclic tetrameric derivative formed in γ-cyclodextrin, has been proved to inhibit proliferation and induce autophagy in breast cancer cells. CTet treatment at a concentration of 4 μM for 8 h significantly enhanced the autophagic lysosomal activity in MDA-MB-231 cells, while that in MCF-7 cells increased slightly. And these effects were attributed to the suppression of Akt activity and the overexpression of p21/cyclin-dependent kinase inhibitor 1A (CDKN1A) and GADD45A [200]. Besides, CTet was found to activate the ER stress response, as well as upregulate recombinant DNA damage-inducible transcript 3 (DDIT3)/C/EBP homologous protein (CHOP) and DDIT4, subsequently inhibiting mTOR expression, leading to the induction of autophagy in breast cancer cells [201]. Abe et al. found a β-carboline alkaloid, harmol, which induces autophagic death in U251MG human glioma cells by suppressing phosphorylation of Akt and mTOR. When an autophagosome is formed, the expression of survivin protein is inhibited, which in turn induces apoptosis, suggesting that harmol-induced autophagy is a pro-apoptotic mechanism [202]. Bisleuconothine A (Bis-A), a novel bisindole alkaloid isolated from the leaves of *Leuconotis griffithii*, has cytotoxic activity against a variety of cell lines. In A549 cells, Bis-A exhibits cytotoxic activity by inducing autophagy without apoptosis. Bis-A triggers the formation of autophagosome but inhibits its degradation via inhibiting the Akt/mTOR signaling pathway, accompanied by the decreased phosphorylation of PRAS40 and p70S6K, increased LC3 lipidation, and upregulated p62 levels [203].

Moreover, the compound Muniziqi granule (MNZQ) is a multi-component herbal preparation for the treatment of diseases caused by endocrine disorders. In melanoma B16 cells, harmine, an active component of MNZQ, inhibits the phosphorylation of Akt and mTOR, as well as increases the expression of LC3-II and p62 protein, thereby inducing autophagic cell death [204]. Another β-carboline alkaloid, flavopereirine, was also found to be involved in the regulation of autophagy in breast cancer cells. Treatment with flavopereirine inhibited the expression level of Akt and translation of mTOR in MDA-MB-231 cells while promoting LC3-II accumulation. Furthermore, flavopereirine-induced LC3-II accumulation was found to be flipped when p38MAPK was inhibited, indicating that flavopereirine can block autophagy in breast cancer cells by regulating the Akt/p38MAPK signaling pathway [205].

### Targeting p62/SQSTM1

The p62, namely sequestosome 1 (SQSTM1), a multifunctional junctional protein, is considered a critical regulator of the autophagic process by directly binding with LC3 to generate autophagosomes [206]. In the process of autophagy, p62 interacts with ubiquitinated protein aggregates through the ubiquitin-associated domain (UBA), and LC3 binds to substrates through the LC3-interacting region (LIR) domain, and then the substrate is transferred into autophagosomes, entering the autophagosomal degradation procedure [207, 208]. At physiological conditions, basal autophagy acts as a signal transduction adapter, and due to the continuous degradation of autophagy, the p62 levels are comparatively low [209]. Defective autophagy, accompanied by insufficient p62/SQSTM1 protein degradation, is common in human tumors. Therefore, p62/SQSTM1 and LC3 are universally used together as the hallmark of autophagic flux in cancer studies [210].

Autophagy, as a selective degradation process, requires a variety of autophagy receptor proteins that can selectively recognize ubiquitinated aggregates and deliver them to the phagophores, which is a prerequisite for autophagic degradation by autophagosomes. p62 is a central regulator of binding to ubiquitinated protein aggregates and processing them into envelopes [211, 212]. Accordingly, the upregulation and/or inefficient degradation of p62 are associated with tumor formation, cancer promotion, and therapeutic resistance [213]. Therefore, it indicates that p62 has the potential to be a therapeutic target for most cancer cells. Racemosin B is a marine alkaloid containing an indolo[3,2-α]carbazole framework, derived from the green alga *Caulerpa racemos*e but with lower biological activities [214]. Therefore, Xiao et al. constructed another active substance, 4-bromo-*N*-(2-(dimethylamino)ethyl)-5,12-dihydroindolo[3,2-a]carbazole-6-carboxamide (25), based on the structure of racemosin B. It was demonstrated that compound 25 significantly suppressed breast cancer cell growth and proliferation, as well as induced cell death via the inhibition of autophagy in breast cancer cells through the accumulation of autophagy-related proteins, like LC3-II and p62 [215].

Manzamine A is an indole alkaloid derived from sponges of *Haliclona* sp., *Xestospongia* sp., and *Pellina* sp. with antitumor, anti-inflammatory, antibacterial, and other biological activities [216]. V-ATPase is an enzyme complex that can use the energy released by ATP hydrolysis to maintain intracellular homeostasis, which can enhance cancer progression and has also been proved to be a potential target for cancer treatment [217, 218]. In this study, manzamine A was found to exhibit strong activity against pancreatic cancer cells. Manzamine A administration results in a sustained accumulation of LC3-II and p62/SQSTM1 in PANC-1 cells. Besides, manzamine A can interfere with the activity of vacuolar v-ATPase in cancer cells and blocks the autophagic pathway by inhibiting the autophagosome turnover and autophagosome-lysosome fusion, thus inhibiting the growth of pancreatic cancer cells [216]. In addition, indole-3-carbinol (I3C), as an indole alkaloidal compound isolated from cruciferous vegetables, reported that upregulating the autophagy markers LC3-II and downregulating the p62 protein expression trigger autophagic cell death in Ehrlich ascites carcinoma cells [219].

### Targeting Beclin-1

Beclin-1, the mammalian orthologue of the yeast ATG6/Vps30, is a key factor that mediates the localization of other autophagic proteins to the pro-autophagosome [220]. The human Beclin-1 gene is encoded by the BECN1 gene, which is located on chromosome 17q21, and its encoded product is a marker for initiating the autophagic process and a key protein for regulating autophagy [221]. Beclin-1 contains three important domains: (1) BH3 domain: it can interact with anti-apoptotic factors like Bcl-2; (2) coiled-coil domain (CCD): it can interact with UV-radiation resistance-associated gene (UVRAG) and PI3KCIII; and (3) evolutionarily conserved domain (ECD): it can bind to PI3KCIII [222, 223]. In the autophagy process, Beclin-1 interacts with multiple interacting vesicles (such as ATG14L, UVRAG, Ambra1, Rubicon, and PINK) to promote the formation of the Beclin-1/Vps34/Vps15 complex. This complex promotes the production of phosphatidylinositol 3-phosphate (PI3P), which in turn stimulates the formation of autophagosomes [224]. Beclin-1 also regulates autophagic activity in cancer by interacting with other autophagic mediators, including ATG, mTOR, p53, and ROS [225].

Recently, Beclin-1 has attracted increasing attention for its antitumor effects, and it has been regarded as a tumor suppressor protein that plays a key role in the lysosomal degradation pathway of autophagy [225, 226]. Gene deletion and reduced expression of Beclin-1 have been found in various cancer cells. In addition, overexpression of the Beclin-1 gene has been reported to suppress tumorigenesis in MCF-7 human breast cancer cells. Studies have shown that Beclin-1 gene deletion is present to varying degrees in 75% of ovarian cancers, 50% of breast cancers, and 40% of prostate cancers [227]. Therefore, Beclin-1-mediated autophagy cell death is an important tumor suppressor mechanism, and Beclin-1 can be studied as a potential target for anticancer therapy. Mukonal, an indole-contained alkaloid derived from *Murraya koenigii*, has shown a potential anticancer effect on breast cancer cells as well as inhibits the proliferation of MDA-MB-231 and SK-BR-3 cells. In mukonal-treated breast cancer cells, there was an increase in the expression level of autophagy proteins, Beclin-1, LC3-I, and LC3-II, thus leading to autophagic cell death. Besides, in vivo experiment indicated that mukonal could significantly reduce tumor weight and volume [228]. Moreover, Beclin-1 was also involved in the evodiamine-induced autophagic death of gastric cancer cells, and its expression level was significantly increased in SGC-7901 cells [229].

### Targeting JAK2/STAT3 pathway

The JAK2/STAT3 signaling pathway is of significance in the regulation of oxidative stress, cellular autophagy, inflammatory response, and tumor progression [182]. STAT3 is a redox-sensitive transcription factor that is involved in the regulation of autophagy when JAK is activated [230]. JAK2 is a non-receptor type tyrosine kinase, and activated JAK2 can induce phosphorylation of reversely activated STAT3, and free STAT3 can dimerize and translocate to the nucleus, which in turn regulates the expression of numerous target genes, including some autophagy-related genes [231]. In malignant tumors, sustained growth signals result in a state of continuous STAT3 activation [232].

Therefore, suppression of the JAK2/STAT3 pathway could shed new light on cancer treatment and afford new targets for tumor therapy. Bufothionine is the main component of cinobufacini injection with an anticancer effect. And a study demonstrates that bufothionine can inhibit proliferation and induce autophagy as a way to exert better anticancer effects [233]. Besides, in bufothionine-treated SMMC-7721 hepatocellular carcinoma cells, it could be observed that the concentration of IL-6 was decreased, the expression of p-Stat3-tyr705, p-Stat3ser727, and JAK2 was downregulated, as well as there was an increase in Atg5, Atg7, and LC3-II. It suggested that bufothionine-induced autophagy in HCC was mediated by suppressing JAK2/STAT3 pathway [233].

### Targeting ROS

ROS are by-products of biological aerobic metabolism, which can balance cell homeostasis under normal physiological conditions, but excessive ROS can cause oxidative stress damage to cells, causing metabolic disorders and inflammatory diseases [234]. Intracellular ROS mainly originate from mitochondria, and mitochondrial autophagy can remove the damaged mitochondria and reduce the overproduction of ROS. Compared to normal cells, cancer cells have higher levels of ROS due to increased metabolism and mitochondrial dysfunction [235].

It has been shown that ROS can participate in the regulation of autophagy as an intracellular signal molecule. In the early stages of tumor formation, mild oxidative stress generates ROS to activate autophagy, which assists normal cells in removing abnormal or damaged organelles and reduces the chance of tumor formation [236, 237]. Elevated ROS triggers autophagy, enhances amino acid recycling, and promotes tumor cell survival and sustained proliferation. When persistent oxidative stress induces a high concentration of ROS, autophagy is overactivated and promotes tumor cell apoptosis [238]. In addition, ROS can also regulate different signaling pathways to induce autophagy. The research conducted by Gos et al. showed that reduced scytonemin (R-scy) isolated from *Nostoc commune* has the competence to inhibit human T-lymphoid Jurkat cell growth, which is caused by autophagic cell death. The R-scy-induced autophagic effect was mediated primarily by large amounts of ROS generation and the subsequent accumulation of LC3-II, resulting in mitochondrial dysfunction [239]. Another alkaloid, fascaplysin, a natural product isolated from the marine sponge, can induce autophagic cell death of leukemia cells by regulating the PI3K/Akt/mTOR pathway [240], and it has also been found that fascaplysin plays an important role in vascular endothelial cells (VECs) autophagy. p8 as a transcriptional factor plays a key role in modulating VECs autophagy. In fascaplysin-treated VECs, the expression levels of p8 protein and ROS were significantly increased, thus stimulating VECs autophagic cell death [241].

### Targeting other autophagic pathways

In spite of the above-mentioned signaling pathways to exert antitumor effects, indole alkaloids can also modulate autophagy through other pathways. Song et al. reported that EE-84, an aplysinopsin derivative, exhibited a potent growth inhibitory effect both in vitro and in vivo. Accordingly, cell autophagy is closely associated with ER stress. EE-84 was shown to trigger ER stress and then promote K562 CML cell autophagy, which is accompanied by morphological changes as well as the activation of the PERK-eIF2α-ATF4-CHOP pathway [242]. These results indicate that EE84 has excellent antileukemic properties and is expected to be a potential drug for the treatment of leukemia. Furthermore, voacamine (VOA), as a natural bis-indole alkaloid derived from the plant *Peschiera fuchsiaefolia*, has been widely reported to enhance the cytotoxic effect of multi-drug resistance (MDR) osteosarcoma cells exposed to doxorubicin (DOX). Subsequently, the researchers found that the chemosensitizing capacity of VOA was mediated by induction of autophagy-dependent cell death rather than apoptosis [243]. LC3, as one of the important genes encoding autophagy-related proteins, is located on the surface of autophagic vesicles, participates in the formation of autophagy, and is recognized as a biomarker of the autophagic process. The ratio of LC3-I to LC3-II is related to the degree of autophagy. When cancer cells were treated with VOA, it was observed that the expression of LC3-II increased in a dose-dependent manner [243]. Overall, VOA, as an autophagy inducer, is a promising therapeutic agent in the treatment of MDR tumors. Zhang et al. isolated and identified 26 alkaloid compounds from *Tabernaemontana corymbosa*, among which Taberine D was a novel vobasinyl-ibogan alkaloid, which exhibited good cytotoxicity to some human cancer cells and attenuated lysosomal acidification. Lysosome acidification blockade leads to lysosome dysfunction, which is related to the interruption of autophagy flux. Besides, the taberine D treatment did not observe a decrease in p62 protein expression, which confirmed that taberine D suppresses autophagy flux [244].

## Targeting other RCD subroutines with indole alkaloids in cancer

Apoptosis and autophagy-dependent cell death are crucial subroutines of RCD, which could induce degradation of organelles or cell death under cellular stress and play a vital role in targeted therapy and regulation of cancer cell death. However, in this review, we not only focus on apoptosis and autophagy-dependent cell death, two classical pathways that regulate cell death, but also involve other RCD subroutines such as ferroptosis, mitotic catastrophe, necroptosis, and anoikis (Table 4).

**Table 4:** Indole alkaloids targeting other RCD subroutines in cancer

Compound	Structure	Source	Target	Mechanism in RCD	Cancer cell line	Tumor type	Refs.
DM10		Bis-isatin derivative	DJ-1, ROS↑	Induce ferroptosis	H1299, MDA-MB-231, BEL7402, 786-O	Lung cancer; breast cancer; liver cancer; renal cancer	[255]
Brucine		*Strychnos nux-vomica*	ATF3, H_2_O_2_, TFR↑ System Xc-↓	Induce ferroptosis	U87 and U251 cells	Glioma	[259]
3,3′-Diindolylmethane		Cruciferous plants	GSH, SLC7A11, GPX4↓ Lipid ROS↑	Induce ferroptosis	BGC-823	Gastric cancer	[260]
Vincristine		*Catharanthus roseus*	CDK1↓ Aurora B, histone-H3, NuMA↑	Induce mitotic catastrophe	HT-29 and HeLa Cells	Colorectal cancer, cervical cancer	[265]
BPR0L075		Combretastatin A-4 derivative	Securin, SAC↑ G2/M phase arrest	Induce mitotic catastrophe	HCT116	Colorectal cancer	[267]
			Cyclin B1, BubR1, MPM-2, survivin↑	Induce mitotic catastrophe	OVCAR-3-TR and SKOV-3-TR	Ovarian cancer	[269]
Staurosporine		*Streptomyces staurosporeus*	RIPK1, MLKL↑	Induce necroptosis	U937 cell	Lymphoma	[275]
10-Methoxycanthin-6-one		Canthin-6-one derivative	RIPK3, MLKL↑	Induce necroptosis	Kasumi-1 cell	Acute myeloid leukemia	[277]
Diindolylmethane		Cruciferous plants	Gli1↓ cleaved PARP, caspase-3↑	Induce anoikis	A2780 and OVCAR-429 cells	Ovarian cancer	[283]
K252a		Staurosporine derivative	BDNF/TrkB↓	Induce anoikis	HONE-1-EBV, HK1-LMP1, C666-1	Nasopharyngeal carcinoma	[284]

↓, downregulate/inhibition; ↑, upregulate/activation

### Targeting ferroptosis

Ferroptosis, an atypical form of RCD, is characterized by iron-dependent oxidative disruption of cellular membranes following the failure of the antioxidant system [245]. The morphological features accompanied by ferroptosis are mainly the decrease in mitochondrial volume, reduction or disappearance of mitochondria crista, and rupture of the outer mitochondrial membrane [246]. Ferroptosis was first proposed by Dixon in 2012 as an iron-dependent form of cancer cell death distinct from apoptosis and autophagy. Ferroptosis is triggered by RAS-selective lethal small molecule called erastin, like glutamate, which could restrain cystine uptake by cystine-glutamate antiporter (system Xc−) and subsequently lead to the exhaustion of glutathione (GSH) and inactivation of the phospholipid peroxidase glutathione peroxidase 4 (GPX4) [247, 248]. Among them, GPX4 and system Xc− have been identified as key players in mediating the onset of ferroptosis and are the main regulatory molecules of ferroptosis. As a matter of fact, iron metabolism and lipid peroxidation are engaged in the process of iron degradation. Polyunsaturated fatty acids (PUFAs) are the raw materials of lipid peroxidation processes that can drive ferroptosis, while the inactivation of GPX4 directly leads to the accumulation of lipid peroxides and subsequently induces ferroptosis [249].

There are various pathways to induce cell death through ferroptosis, among which the two most important trigger mechanisms are: (1) inactivation or inhibition of GPX4: GSH is an important molecule that regulates the oxidative cellular environment, and it is also an essential auxiliary factor of GPX4 [250]. GPX4, a kind of selenium-containing cysteine enzyme, has evolved as a defense mechanism against ferroptosis. It uses reduced GSH to reduce lipid and organic hydrogen peroxide to alcohols and protects cells from lipid peroxide damage. Inactivation of GPX4 by drugs or other means can disrupt the oxidative balance, causing the destruction of cell membrane structure by lipid peroxides, thereby stimulating ferroptosis [251]. (2) Inhibition of system Xc−: some cells take up extracellular cystine mainly through system Xc− and reduce it to cysteine, while cysteine is the raw material for GSH synthesis. Inhibition of system Xc− indirectly leads to depletion of endogenous antioxidant GSH, resulting in the lethal accumulation of ROS and ferroptosis [252]. In addition, alteration of voltage-dependent anion channel (VDAC), modulation of cellular iron concentration, and regulation of the tumor suppressor p53 can also elicit ferroptosis [253]. A variety of indole-contained natural products and synthetic compounds have been demonstrated to be novel ferroptosis regulators and have the potential to treat ferroptosis-related neoplastic diseases. RSL3 is a type of small molecule compound that could suppress the activity of GPX4 to trigger ferroptosis. ROS levels and transferrin expression were enhanced in CRC cells treated with RSL3, accompanied by the GPX4 expression significantly decreased, indicating an iron-dependent cell death and ferroptosis [254]. DJ-1 is a multifunctional protein that is involved in different biological and cellular processes. A series of bis-isatin derivatives with different length linkers have been designed and synthesized to target DJ-1 homodimer, and the compound DM10 with an alkylene chain of C10 exhibits powerful activity. DM10 exerts a remarkable growth inhibition effect on a variety of tumor cells [255]. Moreover, it is known that the deletion of DJ-1 could effectively increase the sensitivity of tumor cells to ferroptosis inducers [256]. The study shows that DM10 markedly amplified the effect of erastin (a ferroptosis activator)-triggered ferroptosis cell death, as well as the accumulation of lipid ROS, which has been monitored by flow cytometry [255].

**Fig. 6:**
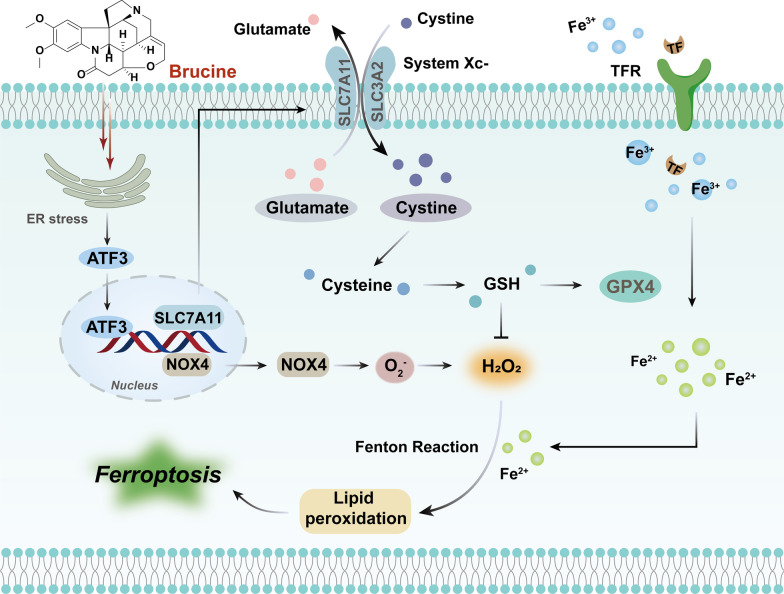
Scheme illustrating the effects of brucine-induced ferroptosis in glioma cell

Disturbance of intracellular iron ion metabolism can lead to ferroptosis cell death. Intracellular iron level is mainly modulated by transferrin receptor (TFR), and the increased expression of TFR will make more Fe^3+^ enter cells and be reduced to Fe^2+^ by iron reductase, which eventually promotes ROS production via Fenton reaction [257]. Activating transcription factor 3 (ATF3) is a common stress sensor and a downstream gene of the ER stress PERK/ATF4 pathway. ATF3 could suppress system Xc− and trigger erastin-induced ferroptosis [258]. Brucine, an indole alkaloid isolated from the seeds of *Strychnos nux-vomica*, was shown to have strong anticancer activity against multiple tumors, including breast cancer, glioma, and colorectal cancer. In a study, brucine was found to suppress the growth of glioma cells in vivo and in vitro [259]. Brucine could upregulate ATF3 levels and promote nuclear translocation through the activation of ER stress. And ATF3 inhibited the activities of catalase and system Xc− to alleviate H_2_O_2_ degradation while upregulating the expression of NADPH oxidase 4 (NOX4) and superoxide dismutase 1 (SOD1) to promote H_2_O_2_ accumulation. In addition, ATF3 facilitated the brucine-induced elevation of Fe^2+^ concentration, upregulation of TFR, as well as lipid peroxidation, which ultimately led to glioma cell ferroptosis [259] (Fig. 6). Another bioactive compound, 3,3′-diindolylmethane (DIM) was shown to induce gastric cancer BGC-823 cells ferroptosis by increasing the accumulation of lipid ROS and decreasing the GSH level. DIM treatment also downregulated the expression of GPX4 and SLC7A11, which are other important ferroptosis regulators. Moreover, the group found that the BAP1–IP3R axis played an important role in exerting anticancer effects against BGC-823 cells, and the activation of IP3R enhanced DIM-induced ferroptosis [260].

### Targeting mitotic catastrophe

Mitotic catastrophe is a type of cell death associated with aberrant mitosis caused by inappropriate or uncoordinated mitotic processes. The well-known morphological features of mitotic catastrophes are multinucleation and micronucleation [261, 262]. According to the NCCD, mitotic catastrophe can constitute a tumor suppressor process that ultimately leads to apoptosis, necrosis, or senescence-mediated elimination of mitosis-deficient and genomically unstable cells [5]. DNA damage, mitotic defects, and cytokinesis failure are the main factors contributing to mitotic catastrophes. Cell cycle progression is controlled by cyclin-dependent kinases (CDKs). CDKs bind to their crucial regulatory subunit named cyclins and drive cell cycle processes [263]. The CDK1/cyclin B1 complex is an indispensable component of mitotic catastrophe, which promotes the transition of the cell cycle from the G2 to M phase, and is involved in chromatin condensation, nuclear membrane destruction, and microtubule recombination [262]. Antitumor drugs that act on microtubules, mitotic spindle, and mitotic checkpoints to treat cancer could induce mitotic catastrophe, while the inhibition of mitotic catastrophe could facilitate the development of chemoresistance and tumorigenesis.

Accordingly, microtubule-targeted agents have been considered effective anticancer drugs in various types of cancer, given their hindrance to mitotic progression. Vinca alkaloids are bis-indole alkaloids found in *Catharanthus roseus*, which could bind to microtubule proteins to accelerate microtubule depolymerization, inhibit microtubule polymerization, hinder the formation of spindle microtubules, and finally arrest cell division at the metaphase of mitosis [264]. Vincristine is an antimitotic microtubule inhibitor, which could induce G2/M phase cell cycle arrest by blocking tubulin polymerization and triggering mitotic catastrophe in cancer cells. In HT-29 cells, after vincristine treatment, it could be obviously observed that the phosphorylation of CDK1 reduced and the phosphorylation of spindle assembly checkpoint (SAC) proteins Aurora B, histone-H3, and nuclear mitotic apparatus (NuMA) increased [265]. Besides, it has been shown that vincristine-induced mitotic catastrophe could overcome the resistance of cancer cells to apoptosis [266]. Nevertheless, due to the poor solubility of vincristine under physiological conditions, its efficacy is lower than that of other small molecules. BPR0L075 (6-methoxy-3-(3′,4′,5′-trimethoxy-benzoyl)-1H-indole) is an analog of Combretastatin A-4 isolated from the South African tree *Combretum caffrum*, and it could be a novel anti-microtubule agent, displayed potent anticancer and anti-angiogenic activities in vivo and in vitro [267]. Securin is a protein that has participated in many biological functions which could regulate sister chromatid separation during mitosis, and it is highly expressed in various tumors to promote tumorigenesis [268]. BPR0L075 could trigger phosphorylation of securing and G2/M arrest, thus destabilizing mitotic regulatory factors to promote mitotic catastrophe in HCT116 cells. Besides, BPR0L075 could also activate the JNK and p38 MAPK pathway to induce cell death [267]. Furthermore, BPR0L075 could induce paclitaxel-resistant ovarian cancer cell death by mitotic catastrophe, with giant multinucleated cells formation and increased levels of cyclin B1, BubR1, MPM-2, and survivin proteins observed [269]. Mitotic catastrophe is becoming increasingly important as a new strategy for the development of novel anticancer agents, especially for the treatment of cancers resistant to conventional chemotherapy.

### Targeting necroptosis

Necroptosis is a subroutine of RCD, and it is performed in response to a specific stimulus and involves the activation of cellular signaling pathways. Necroptosis does not relate to energy metabolism and is a process of cellular volume increasing, organelles swelling, disruption of the membrane integrity, and ultimately activating cell disintegration [270, 271]. Necroptosis depends on the activity of two related kinases, receptor-interacting serine/threonine kinase protein (RIPK) 1 and RIPK3. Specifically, necroptotic cell death is triggered by the TNF family binding to their receptors, which promotes the interaction between RIPK1 and RIPK3, and then activates these kinases and interacts with mixed lineage kinase domain-like protein (MLKL) to form a necrosome complex [272]. RIPK3 phosphorylates MLKL on residues of threonine 357 and serine 358, enhancing its oligomerization, which in turn promotes the transfer of MLKL oligomers from the cytoplasm to the plasma membrane, where it forms pores and disrupts membrane integrity, leading to the occurrence of necroptosis. In addition, in the course of necroptosis, sequential phosphorylation events involving RIPK1, RIPK3, and MLKL are essential for signal transmission. Thus, the activated RIPK1 phosphorylates and activates RIPK3, which in turn activates MLKL through phosphorylation [273, 274].

Necroptosis is closely associated with the development, progression, and metastasis of tumors and may be an important target for the treatment of malignant tumors. Natural compounds have been regarded as an important source for the discovery of novel antitumor agents. Many compounds induce or inhibit necroptosis by targeting crucial factors of the necroptosis pathway, such as RIPK1, RIPK3, and MLKL. Staurosporine is a kinase inhibitor compound isolated from *Streptomyces staurosporeus*, which could trigger apoptosis through the intrinsic pathway, while staurosporine-induced U937 cell death can be considered as necroptosis in the absence of caspase [275]. Under this condition, the inhibition of RIPK1 degradation can be observed to support its role in the staurosporine-triggered necroptosis. Besides, not only RIPK1 but MLKL are also involved in the STS-provoked necroptotic cell death [275]. β-carboline alkaloids are a group of bioactive natural compounds. Canthin-6-one, a natural indole alkaloid derived from several plant genera used as medicines, is reported to be anti-inflammatory, antimicrobial, antitumor, and anti-proliferative [276]. It was reported that canthin-6-one exhibited potent cytotoxicity activity against leukemic Kasumi-1 cells. Herein, it is found that its derivative, 10-methoxycanthin-6-one (Mtx-C), has the highest anticancer activity in the AML Kasumi-1 cells and KG-1 cells, and the mechanism of inducing cell death was established. Mtx-C could promote the necroptotic pathway, accompanied by an increase in phosphorylation of RIPK3 Ser227 and MLKL Ser358 in Kasumi-1 cells [277].

### Targeting anoikis

Anoikis is a specific form of programmed cell death induced by the detachment of cells from ECM or adjacent cells, which plays a vital role in organism development, tissue self-homeostasis, disease development, and tumor metastasis [278, 279]. Whereas, after the cancer cells break away from the adhesion of the ECM and enter the circulatory system, tumor cells do not undergo apoptosis and regain the adhesion ability to spread, metastasize and invade, which is called anoikis resistance [280]. In particular, some malignant tumor cells, which are prone to distant metastasis, display extremely strong anoikis resistance properties [278]. Resistance to anoikis is a main characteristic of cancer cells that undergo metastasis, and it is also a key reason for treatment failure and patient death.

In spite of its unique induction, the mechanism of anoikis is quite similar to that of apoptosis, and their pathways share multiple similarities, following either the intrinsic or the extrinsic pathway [281]. Targeting anoikis to regulate cancer cell growth and metastasis has become a promising therapeutic strategy for the development of natural compounds. Gli1 is an essential transcription factor of the hedgehog pathway, which is aberrantly expressed in various tumors. Studies have shown that the hedgehog pathway plays a role in cell cycle progression, angiogenesis, and metastasis by regulating Gli1 expression [282]. In addition, it is reported that DIM can reduce anoikis resistance by inhibiting the expression of Gli1. In A2780 and OVCAR-429 cells, silencing Gli1 expression could induce the cleavage of PARP [283]. Sonic hedgehog (Shh) is one of the family proteins of hedgehog signaling [282], which could block the decrease in Gli1 expression mediated by DIM, thus inducing the occurrence of anoikis. Therefore, it can be considered that the hedgehog pathway plays a major role in anoikis resistance. In addition, when ovarian cancer cells are treated with a hedgehog inhibitor, cyclopamine, and Gli1 siRNA, the anoikis resistance can be significantly reduced, substantiating that Gli1 is a key protein in the induction of anoikis in ovarian cancer [283]. Nasopharyngeal carcinoma (NPC) is a highly aggressive and metastatic head and neck cancer that is closely related to Epstein-Barr virus (EBV) infection. It is found that the brain-derived neurotrophic factor (BDNF)/tropomyosin-related kinase B (TrkB) pathway plays a vital role in regulating metastasis and anoikis resistance of NPC cells [284]. The research employed EBV-related NPC cell lines, HONE-1-EBV, HK1-LMP1, and C666-1 cells and demonstrated that K252a, a staurosporine analog, is also a Trk inhibitor which could inhibit the migration as well as the proliferation of cancer cells. In addition, K252a was shown to significantly attenuate anoikis resistance in NPC cells by inhibiting the expression of BDNF and TrkB [284]. In a word, K252a has a promising pharmacological targeting effect and can be used as an anoikis sensitizer for NPC, preventing metastatic tumor cells from surviving in the isolated state.

## Key signaling involved in cross talk between different RCD subroutines modulated by indole alkaloids

Interestingly, cell death pathways are not isolated linear signaling cascades as depicted in the schematic diagram. In contrast, the pathways modulating different patterns of cell death are interconnected at multiple levels [285]. As an important intracellular material degradation pathway in eukaryotes, autophagy can wrap proteins and organelles into autophagosomes and fuse with lysosomes to degrade them, which is an important mechanism for maintaining cellular homeostasis [286]. Apoptosis and autophagy are two important forms of regulated cell death, which play an essential role in the development of tumors [287]. Meanwhile, there is abundant evidence that apoptosis and autophagy do not exist in isolation, and some key proteins frequently appear in the regulatory network of both, indicating that there is a complex interaction between them, and they can promote cell death through independent or complementary relationships [288, 289].

A variety of antitumor drugs can not only contribute to the onset of apoptosis but also induce autophagy in cancer cells, and it has been suggested that autophagic death of tumor cells occurs due to excessive autophagy [290, 291]. It is of great significance to explore the relationship between apoptosis and autophagy induced by natural indole alkaloids for tumor therapy and novel antitumor drug discovery.

The MAPK signaling pathway is an essential cross talk mediator in the regulation of apoptosis and autophagy, including ERK1/2, JNK1/2, and p38 MAPK. CAA45, a novel semisynthetic derivative of calothrixin A (CAA), was capable of inducing mitochondria-mediated cell apoptosis and promoting autophagy in non-small cancer cell A549 cells with concomitant inhibition of Akt, activation of JNK and p53, and aggregation of LC3-II. Besides, CAA45 can suppress Topo I activity, block the cell cycle at the S phase, as well as inhibit the migration of A549 cells by downregulating the expression level of MMP-2 and MMP-9 [102]. Autophagic cells are closely associated with the onset of apoptotic tumor cell killing. It was reported that dimeric β-carbolines displayed potent cytotoxicity in multiple cancer cell lines, and in NSCLC cells, dimeric β-carboline 1 accumulated in lysosomes and suppressed autophagy, and then upregulated the expression of Puma, thus leading to the induction of caspase-dependent apoptotic cell death [292]. Recently, flavopereirine was shown to exert antigrowth activity against HepG2 and Huh7 cells, possibly through the induction of G0/G1 cell cycle arrest, as well as triggering apoptotic and autophagic cell death [293].

There are multiple molecular mechanisms involved in regulating the cross talk between autophagy and apoptosis, with the interaction and modification of Beclin-1 and Bcl-2 at the expression level playing a key role [294]. Furthermore, there is growing evidence that autophagy and apoptosis, two crucial processes that regulate cell death, interact and influence each other, often through the PI3K/Akt/mTOR pathway, NF-κB pathway, and Bcl-2 family proteins contributing to cross-functional mutual regulation [287]. In the human NSCLC epithelial A549 cells, ellipticine, a topoisomerase II inhibitor, exhibited potent cytotoxicity and anti-proliferation effects, which were mediated by the induction of apoptotic and autophagic death via promoting the nucleus translocation of p53 and phosphorylated Akt, as well as the recruitment of autophagosomes to elicit the development of autophagic cells [295]. BMA-155Cl, a novel bisindolylmaleimide alkaloid, was shown to possess a pro-autophagic and pro-apoptotic effect on human hepatocarcinoma HepG-2 cells in vivo and in vitro. Following the administration of BMA-155Cl, the protein expression levels of Beclin-1, LC3-II, NF-κB p65, p53, and Bax were correspondingly increased in a dose-dependent manner, while the IκB and Bcl-2 levels were decreased, indicating that NF-κB p65 pathway plays a vital role in regulating apoptosis and autophagy. In addition, BMA-155Cl significantly inhibited the growth of HepG-2 xenograft tumors in vivo [296].

In addition, caspases have been observed to be direct players in the cross talk between apoptosis and autophagy. Activated caspases are converged by multiple pro-apoptotic signals that activate caspases to launch apoptosis and degrade important autophagy-related proteins such as Beclin-1, ATG5, and ATG7 [297, 298]. Fascaplysin is a marine sponge-derived alkaloid that induces cell cycle arrest at the subG1 phase and promotes autophagy and apoptosis in human leukemia HL-60 cells in vivo and in vitro, with the two pathways synergistically promoting cancer cell death [240]. Among them, fascaplysin-induced apoptosis can be mediated by stimulating PARP-1 cleavage and caspase activation, whereas autophagy is triggered by increasing the expression of LC3-II, ATG7, and Beclin-1 genes. Interestingly, caspase-dependent apoptosis and autophagy induced by fascaplysin were also closely associated with inhibition of the PI3K/Akt/mTOR pathway, which provides further insight into its anticancer effects [240]. Angiogenesis is a hallmark manifestation of tumor growth and development [299]. Subsequently, the group designed a fascaplysin derivative, 4-CF, which has anticancer and anti-angiogenic effects. In addition to affecting the expressions of some pro-angiogenic factors such as HIF-1α, eNOS, and MMP-2/9, 4-CF can also inhibit the survival of human umbilical vascular endothelial cells and breast cancer cells by inhibiting the PI3K/AKT/mTOR pathway. Moreover, 4-CF could significantly trigger both autophagy as well as apoptosis via the changes in their biomarkers, like LC3-II, caspase-3, and PARP-1 [300]. Violacein (VIO), an indole derivative isolated from Gram-negative bacteria species, inhibits cell growth by inducing apoptosis and autophagy in head and neck carcinoma cell lines, which was associated with ROS generated, NF-κB and the Bax/Bcl-2 ratio upregulated, and the expression of p53 degraded. Beclin-1 is a protein that promotes autophagy, and when Bcl-2 binds to Beclin-1, it inhibits autophagy. VIO can reduce Bcl-2 expression in HNC cells, thereby activating Beclin-1 to induce autophagy [301]. Chaetocochin J has strong anti-proliferation activity against CRC cells and can simultaneously induce apoptosis and autophagy of CRC cells. The response to these effects is facilitated by regulating AMPK and PI3K/Akt/mTOR pathways [302]. Moreover, in gastric cancer MGC803 and SGC-7901 cells, harmine simultaneously elicits apoptosis and autophagy to exert anti-proliferation activity. Harmine-induced autophagy is mediated by inhibition of the Akt/mTOR/p70S6K signaling pathway, which is accompanied by increased expressions of Beclin-1, LC3-II, and p62; and the AMPK pathway also participates in regulating the autophagy process. The occurrence of apoptosis is related to mitochondrial-mediated signaling pathways, including regulating the expression of Bcl-2 family proteins and caspase cascade [303].

Furthermore, according to the phenomenon of multi-drug resistance in chemotherapy, studies have found that the most effective method to resist multi-drug resistance is to simultaneously activate different cell death pathways such as apoptosis, autophagy, and necroptosis [304]. In this study, isomahanine has shown significant cytotoxicity to CLS-354/DX cells (a multi-drug-resistant OSCC cell line) by inducing both caspase-dependent apoptosis and autophagy which was followed by LC3-II expression increased and p62 degraded. Besides, isomahanine-induced ER stress and activation of the p38MAPK pathway also mediate apoptosis and autophagic cell death [305]. Multitarget drugs are usually composed of two or more active pharmacodynamic structures, which can target multiple signaling pathways and targets, thereby enhancing the biological activity and therapeutic effect of drugs [306]. Ling et al. proposed to introduce hydroxamic acid functional groups (the active fragments of the HDAC inhibitor, SAHA) into β-carboline to generate novel hydroxamic-acid-containing β-carboline compounds. These compounds showed better cytotoxic effects on most cancer cells, including drug-resistant Bel7402 cells. Apoptotic and autophagic cell death of Bel7402 cells were triggered by modulating the activity of apoptosis-related proteins, like Bax, Bcl-2, and caspase-3, and the expression of autophagic fluxes, like Beclin-1, LC3-II, and p62. In addition, inhibition of the PI3K/Akt/mTOR pathway can also promote cell death [307]. More research is needed in the future to focus on the complex interactions among cell death signaling pathways and drug resistance mechanisms. Heat shock protein 70 (Hsp70) is a highly conservative molecular chaperone that plays a key role in maintaining cell homeostasis [308]. Hsp70 is overexpressed in a large variety of different tumor types and provides malignant tumor cells a selective advantage by interfering with tumor immunity and promoting angiogenesis, cell proliferation, and cell invasion [309]. Hsp70 can also interact with Apaf-1 and apoptosis-inducing factor 1 (AIF1) to exert an anti-apoptotic effect [310]. Vincristine can block the Hsp70 binding with Sirt2 to induce mitochondrial autophagy, leading to the acetylation of Hsp70 at K126, which in turn inhibited Hsp70-mediated invasion and migration of breast cancer cells, as well as stimulated mitochondria-mediated apoptosis in MDA-MB-231 cells [311].

Apoptosis and necroptosis are interconnected rather than completely separated, and both forms of cell death can be observed in some pathological tissues simultaneously [312]. Staurosporine is a potent inducer that can induce necroptosis in rat astrocytes at high doses in addition to inducing apoptosis. The induction of necroptosis is closely mediated by the activation of RIPK1 activity, independent of caspase response [313]. RIPK3 acts as a downstream regulator of RIPK1, and MLKL is a crucial protein in the activation of necroptosis [314]. 10-methoxycanthin-6-one (Mtx-C) is a derivative of indole-containing natural canthin-6-one alkaloid, which exhibits potent cytotoxicity to AML Kasumi-1 and KG-1 cells. After administration of Mtx-C, the expression of apoptosis-related proteins Bax, Bim, Bik, Puma, and p53 was upregulated, thereby promoting the induction of apoptotic cell death. In addition, Mtx-C can regulate the necroptotic pathway, which is associated with phosphorylation of RIP3 Ser227 and MLKL Ser358. Meanwhile, DNA damage and activation of p38 and JNK MAPKs stress pathways are also involved in Mtx-C-induced cell death [277].

Moreover, under certain conditions, activated necroptosis can be used as an alternative cell death for apoptotic pathway, making it a potential therapeutic target in apoptotic resistant cells [315]. 11-methoxytabersonine (11-MT) was reported to strongly induce necroptosis as well as stimulate autophagy, thus providing a cytoprotective effect against necroptosis. 11-MT strengthened the interaction of RIP1 with RIP3, thus promoting the formation of necrosome in A549 and H157 cell lines. Besides, it was found that the induction of autophagy was mediated by regulating the AMPK, mTOR, and JNK signaling pathways [316]. And this natural indole alkaloid can be effectively enhanced to kill cancer cells that are resistant to other apoptosis-inducing chemotherapeutic agents.

## Combination therapies of indole alkaloids

Combined therapy, due to the fact that it targets multiple signaling pathways, a variety of mechanisms can be utilized to mitigate the development of resistance to antineoplastic drugs. MDR refers to the fact that after long-term exposure to a certain antitumor drug, tumor cells will develop resistance to the drug and cross-resistance to other antitumor drugs with different structures and functions, which is one of the main reasons for the failure of tumor chemotherapy. The emergence of MDR was a major obstacle to cancer disease treatment [317]. Some new combination therapy strategies can reverse chemotherapy resistance in cancer to some extent. Targeted therapies using natural products in combination with existing anticancer drugs appear to be more effective than single-drug therapies in the treatment of cancer [318]. Meanwhile, the combined action of anticancer drugs with natural products provides synergistic action and helps to ameliorate the overall therapeutic effect against cancer cells. The interaction of classical chemotherapeutic agents with natural substances introduces a new perspective to cancer exploration and treatment, representing a future research direction [319]. Moreover, some targeted therapies combined with immunotherapy may exert more powerful antitumor effects. Immunotherapy utilizes the immune system to suppress cancer development by stimulating antitumor immune responses [320]. RCD can participate in the survival, differentiation, activation, and transport of immune cells, thus improving the efficacy of immunotherapy, overcoming immunotherapy resistance, and inhibiting tumor growth by modulating tumor immune response. Furthermore, it has been shown that RCD subroutines can exhibit not only synergistic antitumor immune responses, but also exert an inhibitory effect on antitumor immune responses [321]. Hence, combination therapy may be a viable strategy to improve the treatment efficacy (Table 5). The marine alkaloid 3,10-dibromofascaplysin (DBF) displayed strong cytotoxicity against human prostate cancer cells, and DBF could induce apoptotic death in drug-resistant prostate cancer cells. When DBF was combined with the PARP inhibitor, olaparib, it had a synergistic inhibitory effect on cancer cells, which may be caused by the accumulation of ROS [322]. In addition, the combination of DBF, a strong inducer of apoptosis, and cytarabine can cause pronounced synergistic cytotoxic effects on different myeloid leukemia cells in vitro and finally inhibit the growth of leukemia cells [152]. As mentioned above, EE-84, as an aplysinopsin derivative, has potential antileukemic effects. In order to enhance its anticancer activity, the combination of EE-84 and the Mcl-1 inhibitor, A-1210477, was shown to cause apoptotic cell death in imatinib-sensitive and resistant K562 cells and activate the caspase cascade, producing a synergistic cytotoxic effect [242]. This combination method provides a research basis for the treatment of drug-resistant leukemia. In a study, when harmine derivative, compound 3c, and PI3K inhibitor, LY294002, were combined, they could synergistically inhibit the phosphorylation of Akt and promote the cleavage of PARP and caspase-3, enhancing the apoptosis-inducing effect of compound 3c in CRC cells [133]. Studies have shown that brucine, a cytotoxic drug, can exert strong effects in the combined treatment of cancer. When brucine was treated with gemcitabine in MCF-7 breast cancer cells, the results showed that they could synergistically inhibit cancer cell growth and migration, as well as brucine also inhibited the expression of NF-κB p65 in MCF-7 cells [323].

**Table 5:** Combination therapy for indole alkaloids to treat cancer

Compound 1	Compound 2	Mechanism in RCD	Tumor type	Refs.
3,10-Dibromofascaplysin	Olaparib (PARP inhibitor)	Induce apoptosis	Prostate cancer	[322]
	Cytarabine (DNA synthesis inhibitor)	Induce apoptosis	Myeloid leukemia	[152]
EE-84	A-1210477 (Mcl-1 inhibitor)	Induce apoptosis	Chronic myeloid leukemia	[242]
1-(4-Methoxystyryl)-2-benzyl-9-(3-phenylpropyl)-β-carbolinium bromide	LY294002 (PI3K inhibitor)	Induce apoptosis	Colorectal cancer	[133]
Brucine	Gemcitabine (DNA synthesis inhibitor)	Induce apoptosis	Breast cancer	[323]
Indole-3-carbinol	Hydroxychloroquine (Antimalarial agent)	Induce apoptosis and autophagy	Ehrlich ascites carcinoma	[219]
Indole alkaloid derivative B	5-Fluorouracil (Thymidylate synthase inhibitor)	Induce autophagy	Lung cancer; breast cancer; colonic adenocarcinoma	[325]
Brassinin	Capsaicin (Chilli pepper)	Induce apoptosis	Prostate cancer	[326]
Jerantinine B	*δ*-Tocotrienol	Induce apoptosis	Glioblastoma, colorectal cancer	[328]
Jerantinine A	*γ*-Tocotrienol	Induce apoptosis	Brain cancer	[329]
Vincristine	LY294002 (PI3K/Akt inhibitor)	Induce apoptosis	Gastric cancer	[331]
	Berberine (*Coptidis rhizome*)	Induce apoptosis	Hepatocellular carcinoma	[332]
	Cotylenin A (plant growth regulator)	Induce apoptosis and autophagy	Multiple myeloma	[333]
Vinblastine	Temsirolimus (mTOR inhibitor)	Induce apoptosis	Hepatocellular carcinoma	[334]
	Indibulin (Microtubule inhibitor)	Induce apoptosis	Breast cancer	[335]
	Indirubin (*Indigo naturalis*)	–	Cervical cancer	[336]
Vinorelbine	TRAIL (Apoptosis inducer)	Induce apoptosis	Non-small cell lung cancer	[339]
	FGF401 (FGFR-4 inhibitor)	Induce apoptosis	Hepatocellular carcinoma	[342]
	Lenvatinib (Tyrosine kinase inhibitor)	Induce apoptosis	Anaplastic thyroid cancer	[343]
	Schisandrin B (*Schisandra chinensis* (Turcz.) Baill.)	Induce apoptosis	Gastric cancer	[345]

In addition to inducing autophagic death of cancer cells by regulating the expression of autophagy-related markers p62 and LC3-II, I3C, when used in association with HCQ, can induce apoptosis of EAC cells by activating cleaved caspase-3 and Bax while inhibiting the Bcl-2 protein level, thus prolonging the survival time of mice. The study showed that the combination of I3C with chemotherapeutic agents exhibited better antitumor activity by promoting autophagic and apoptotic cell death than did I3C alone [219]. Cisplatin is also a first-line chemotherapeutic agent commonly used in cancer treatment, but long-term use will cause the development of drug resistance, which eventually leads to cancer recurrence [324]. Furthermore, a novel indole alkaloid derivative B (IADB) has shown to attenuate the cardiotoxic effects caused by the chemotherapeutic drug 5-FU, and the combined application of IADB and chemotherapy can decrease the dose of anticancer agents, improve chemotherapeutic efficacy, and reduce off-target effects to some extent. It was also shown that IADB could induce autophagic cell death of tumor cells, which is mediated by the large amounts of ROS production, thus exhibiting its cardioprotective effects [325]. Brassinin (BSN) is an indole nucleus-containing phytoalexin derived from the cruciferous vegetable; capsaicin (CAP) is an alkaloid extracted from chili pepper. Both natural compounds have been shown to possess anticancer activity against many kinds of tumor cells. Treatment of prostate cancer PC-3 cells with BSN and CAP at the same time showed a synergistic antitumor effect by inducing early and late cell apoptosis, accompanied by the expression of Bcl-2, survivin, cyclin D1, c-Myc, and COX-2 proteins decreased and the expression of p53 protein increased [326]. Jerantinine B is a natural indole alkaloid with strong anticancer activity, but at high doses, it may be cytotoxic to normal cells and cause adverse effects [99]. And tocotrienol was shown to possess the anticancer property but with limited bioavailability and therapeutic responsiveness [327]. Therefore, the low-dose combination therapy strategy is utilized to reduce side effects without reduced therapeutic efficacy. Studies in this group showed that the synergistic inhibitory effect of jerantinine B and δ-tocotrienol on U87MG and HT-29 cells at low doses could be observed, and the characteristics of cell apoptosis and DNA double bond-breaking were also exhibited [328]. Furthermore, jerantinine A combined with γ-tocotrienol at lower concentrations could minimize the toxicity of normal MRC5 cells while enhancing the toxic effect on brain cancer U87MG cells. This combination could elicit apoptosis via triggering the Fas-mediated death receptor pathway and p53-induced mitochondrial pathway [329].

Vinca alkaloids and their derivatives, either alone or in combination with therapeutic drugs, have long been used in the treatment of various types of cancer. Vincristine (VCR) is a well-known antitumor drug, but its clinical application is limited due to its cytotoxicity and drug resistance. The anticancer activity of vincristine could be greatly improved by its co-administration with other drugs providing more enhanced activity with fewer side effects [330]. PI3K/Akt inhibitor, LY294002, synergizes with VCR to promote the growth inhibition of gastric cancer cells and inhibit tumor invasion and migration, which in turn induces apoptosis, and increases intracellular drug accumulation, as well as improves cancer cell sensitivity to VCR. The drug resistance of tumors can be ameliorated by inhibiting the activity of the PI3K/Akt signaling pathway, thus providing a new strategy for MDR therapy [331]. The combined application of VCR and berberine can also dramatically inhibit the growth of HCC cells as well as enhance the ability of apoptosis, which is related to mitochondrial dysfunction and ER stress induction, indicating that berberine is a powerful adjuvant anticancer drug. Besides, berberine can also inhibit the transcription of a drug-resistant gene, MDR-1 [332]. A plant growth regulator, cotylenin A, and VCR could synergistically suppress the growth of myeloma cells and effectively inhibit the formation of large colonies. In addition, combined treatment with cotylenin A and VCR preferentially induce apoptosis as well as trigger autophagy by promoting the formation of LC3-II and the degradation of p62 [333].

Temsirolimus, as a selective mTOR inhibitor, is used to effectively suppress the growth of cancer cells. The study shows that simultaneous targeting of microtubule and mTOR can reduce microvessel density (MND) in Huh7 and Hep3B models. Moreover, temsirolimus/vinblastine combination treatment specifically inhibits the expression level of survivin, Bcl-2, Mcl-1, etc., resulting in the occurrence of apoptosis and the decreasing of survival activity in HCC cells [334]. Indibulin, an indole-contained microtubule inhibitor, has shown better anticancer activity in phase I clinical trials. In breast cancer cells, administration of indibulin could reduce interkinetochore tension, activate the Mad2 and BubR1 proteins, and trigger a mitotic arrest. When it is used in association with vinblastine, it has a synergistic cytotoxic effect on MCF-7 cells [335]. Indirubin is a natural bis-indole alkaloid with high activity, which has significant antitumor activity against CML and is well tolerated in vivo. In addition, vinblastine, as a microtubule-targeting drug, was introduced into indirubin, and it revealed a strong synergistic effect between them, which could block mitosis and inhibit the proliferation of HeLa cells [336]. The above viewpoints further demonstrate that vinblastine can play a marked antitumor effect alone or in combination.

It has been reported that NSCLC is insensitive to the conventional cisplatin/paclitaxel chemotherapy regimens in clinical practice, and it is necessary to develop some new therapeutic strategies to change the status quo. Vinorelbine is a semisynthetic vinca alkaloid that has been used as a single agent or in association with other agents (platinum or gemcitabine) to treat NSCLC in some adjuvant and advanced therapies with favorable results [337]. In addition, it was shown that combining chemotherapy drugs with TRAIL is a means to treat tumors. TRAIL can improve the sensitivity of chemotherapeutic agents by triggering apoptosis, but it has no toxicity to normal cells, which is regarded as a promising antitumor drug [338]. Zhu et al. combined vinorelbine and TRAIL to treat the A549 cell and BALB/c nude mice model and found that the level of tumor growth inhibition and apoptosis induction was stronger. The combination of the two drugs could further upregulate the expression level of apoptosis-related proteins such as Bax and caspase-3 to inhibit the growth of lung cancer cells [339]. Fibroblast growth factor 19 (FGF19) is a driver oncogene that is amplified in approximately 14% of HCC patients and overexpressed in 50% of HCC patients, making the FGF19 gene a novel potential target for the treatment of HCC [340, 341]. Combined treatment of FGF401 and vinorelbine can synergistically inhibit the growth of HCC tumor models with high expression of FGF19 and promote cell apoptosis, thereby prolonging the overall survival of mice and improving the antitumor activity [342]. Recently, the combination of vinorelbine and lenvatinib showed a strong synergism in anaplastic thyroid cancer cells, associated with modulating ABCB1 transporter, reduced CSF-1 expression, with an inhibition of Akt/GSK3β/PRAS40 pathway, and a decrease in the phosphorylation of Src. Besides, the concomitant treatment of vinorelbine and lenvatinib decreases the tumor size of the subcutaneous tumor model [343]. Functional liposomes have high targeting delivery and sustained release properties, which can substantially reduce drug aggregation in non-targeted sites and decrease the toxic side effects of drugs [344]. Li et al. proposed that schisandrin B- and vinorelbine-coated liposomes could be used to inhibit cancer cell metastasis as an effective antitumor agent. In in vitro experiments, the results showed that the targeting liposomes could trigger apoptosis of BGC-823 cells and inhibit tumor metastasis, which is mediated by downregulating the expressions of VEGF, VE-CaD, HIF-1a, PI3K, MMP-2 and FAK [345].

As is known to all, regulation of tumor microenvironment is conducive to the improvement in antitumor efficiency. Activated CD8+ T cells have an antitumor immune effect in a variety of tumors, and when PD-1 is combined with PD-L1, the activation, expansion, and effector function of CD8+ T cells can be inhibited so as to help tumor cells escape immune destruction, thus promoting tumor survival [346]. Thus, in the Lewis lung cancer model, evodiamine exhibited strong antitumor activity by increasing the expression of CD8+ T cells in vivo. In addition, when combined with anti-PD-1 monoclonal antibody (mAb), evodiamine can effectively inhibit tumor growth and prolong the survival time of mice [347], suggesting that evodiamine could be used in conjunction with immunotherapeutic methods to treat NSCLC.

## Conclusions and future perspectives

Regulated cell death is the most important form of cell death, limiting the survival as well as the spread of malignancies. In addition to the typical apoptosis and autophagy, RCD is also involved in multiple death procedures, including ferroptosis, mitotic catastrophe, necroptosis, anoikis, etc. Exploring the role of RCD in anticancer therapy will contribute to our better understanding of cancer pathogenesis, mastering the key targets for controlling cell death, and formulating appropriate therapeutic strategies [348, 349]. Accordingly, it has become the most widely discussed topic in cancer treatment and aroused the interest of most researchers. Under certain circumstances, there is considerable cross talk among RCD subroutines such as apoptosis, autophagy, and necroptosis, and these regulatory mechanisms may show interplay. Thus, targeting molecular targets with related anticancer drugs at their intersection would eventually induce changes in the fate of cancer cells. In short, simultaneous regulation of two or more subroutines of RCD could be a promising strategy for cancer therapy. There is extensive interaction between tumor cell death mechanisms and immune system activation, and targeted therapies play an important role in improving the effectiveness of immunotherapy. In the future, we aim to carry out more combination therapies that combine targeted therapies with radiotherapy, chemotherapy, and immunotherapy, which are essential to improve the therapeutic effect and balance the adverse reactions. The determination of cell death is regulated by a number of factors, including energy levels, DNA damage or stress, and inhibition of specific pathways and targets [350]. A growing body of evidence has delineated that many indole alkaloids regulate apoptosis, autophagy-dependent cell death, ferroptosis, mitotic catastrophe, and other RCD subroutines to eliminate cancer cells, underscoring the key importance of RCD in fighting cancer [7]. Along with the development of medicine and pharmacy, we can utilize natural indole alkaloids such as vinblastine, vincristine, and reserpine as lead compounds, and through the computer-aided drug design (CADD), to synthesize more and more small molecule compounds with novel scaffold, and through further screening and optimization, to develop new anticancer drugs with high efficiency, strong selectivity, and new action targets.

Indole alkaloids and their derivatives have been recognized as important target molecules in medicinal chemistry and drug development, which are abundant in a wide variety of natural sources. However, several indole alkaloids are limited in clinical applications due to their own reasons, such as poor water solubility, low bioavailability, and toxic side effects, and most studies have been conducted in vivo or in vitro using animal models. It is the existence of these drawbacks that can better motivate researchers to investigate novel drugs and promote better small molecule compound synthesis so as to obtain better safety indicators, improved pharmacological properties, and drug effectiveness. If some special processes are used to prepare nanoparticles, liposomes, microspheres, cyclodextrin inclusions, etc., the bioavailability of drugs may be improved, and the side effects would be reduced [351]. Researchers have developed a liposomal co-delivery of polymeric micelle followed by a combination of vinorelbine and cisplatin and found that this liposome has a synergistic antitumor effect and obvious efficacy in reducing tumor size [352]. In addition, evodiamine was combined with a wood oil emulsive nanosystem to improve the solubility of the drug and the sensitivity of cancer cells to evodiamine [353]. To some extent, different drug delivery systems have solved the problems of poor drug solubility and low bioavailability, which is a promising therapeutic tool and offers new perspectives and possibilities for tumor treatment.

In this review, we provide a detailed description of the mechanism of indole alkaloids targeting RCD subroutines and their potential biological activity in the prevention and treatment of cancer. By revising the preclinical studies, we have demonstrated that indole alkaloids can serve as an excellent drug precursor; on this basis, we design new anticancer drugs with higher activity and assist in the improvement in current therapeutic strategies. More recently, the search for optimized or simplified indole alkaloids has become a hot topic of anticancer drug discovery.

## Data Availability

Not applicable.

## Abbreviations

ACD: Accidental cell death
AIF1: Apoptosis-inducing factor 1
Akt: Protein kinase B
AML: Acute myelocytic leukemia
AMPK: Adenosine 5′-monophosphate-activated protein kinase
Apaf-1: Apoptotic protease activating factor 1
ARTS: Apoptosis-related protein in the TGF-β signaling pathway
ATF3: Activating transcription factor 3
ATG: Autophagy-related genes
BARD1: BRCA1-associated ring domain protein
Bcl-2: B cell lymphoma 2
Bcl-xL: B cell lymphoma-extra-large
BDNF: Brain-derived neurotrophic factor
BH3: Bcl-2 homology domain 3
BTK: Bruton's tyrosine kinase
CADD: Computer-aided drug design
cAMP: Cyclic adenosine monophosphate
CCD: Coiled coil domain
CDK1: Cyclin-dependent kinase 1
CDKN1A: Cyclin-dependent kinase inhibitor 1A
cGMP: Cyclic guanosine monophosphate
CHOP: C/EBP homologous protein
CLL: Chronic lymphocytic leukemia
CML: Chronic myeloid leukemia
COX-2: Cyclooxygenase-2
CRC: Colorectal cancer
CREB: cAMP response element-binding protein
Cyt-C: Cytochrome C
DDIT3: DNA damage-inducible transcript 3
DED: Death effector domain
DIABLO: Direct IAP-binding protein with low pI
DISC: Death-inducing signaling complex
DNA: Deoxyribonucleic acid
DR: Death receptor
DSB: Double-strand break
EBV: Epstein–Barr virus
EC50: Median effective concentration
ECD: Evolutionarily conserved domain
ECM: Extracellular matrix
EGFR: Epidermal growth factor receptor
ER: Endoplasmic reticulum
ERK: Extracellular signal-regulated kinase
FADD: Fas associated via death domain
FasL: Fas ligand
FDA: Food and Drug Administration
FGF19: Fibroblast growth factor 19
FIP200: Focal adhesion kinase interacting protein of 200 kD
FLT-3: FMS-like tyrosine kinase 3
FOXO3: Forkhead box O 3
GC: Gastric cancer
GnRHR: Gonadotropin-releasing hormone receptor
GPXa: Glutathione peroxidase 4
GSCs: Glioma stem cells
GSH: Glutathione
HBP: Hamster buccal pouch carcinogenesis
HCC: Hepatocellular carcinoma
HDR: Homologous DNA repair
HR: Homologous recombination
Hsp70: Heat shock protein 70
IC_50_: Half maximal inhibitory concentration
JAK: Janus kinase
JNK: C-Jun N-terminal kinase
LC3: Light chain 3
LEF: Lymphatic enhancer factors
LIR: LC3-interacting region
mAb: Monoclonal antibody
MAPK: Mitogen-activated protein kinase
Mcl-1: Myeloid cell leukemia-1
MDR: Multi-drug resistance
MK2: MAPK-activated protein kinase 2
MLKL: Mixed lineage kinase domain-like protein
MMP: Matrix metalloproteinase
MND: Microvessel density
MOMP: Mitochondrial outer membrane permeability
mTOR: Mammalian target of rapamycin
NCCD: Nomenclature Committee on Cell Death
NF-κB: Nuclear factor kappa-B
NOD1: Nucleotide-binding oligomerization domain 1
NOX4: NADPH oxidase 4
NPC: Nasopharyngeal carcinoma
NSCLC: Non-small cell lung cancer
NuMA: Nuclear mitotic apparatus
OSCC: Oral squamous cell carcinoma
PARP: Poly-ADP-ribose polymerase
PDE3: Phosphodiesterase 3
PI3K: Phosphatidylinositol 3 kinase
PI3KCIII: Phosphatidylinositol 3 kinase complex III
PI3P: Phosphatidylinositol 3-phosphate
PTEN: Phosphatase and tensin homologue deleted on chromosome ten
PUFAs: Polyunsaturated fatty acids
RCD: Regulated cell death
RIPK: Receptor-interacting serine/threonine kinase protein
ROS: Reactive oxygen species
SAC: Spindle assembly checkpoint
SARs: Structure–activity relationships
SMAC: Second mitochondria-derived activator of caspase
SOD1: Superoxide dismutase 1
SQSTM1: Sequestosome 1
STAT3: Signal transducer and activator of transcription 3
T-ALL: T cell acute lymphoblastic leukemia
TAZ: PDZ-binding motif
TCF: T cell factors
TFR: Transferrin receptor
TGF-β: Transforming growth factor-β
TNF-α: Tumor necrosis factor-α
TNFR1: Tumor necrosis factor receptor 1
TRADD: TNFRSF1A associated via death domain
TRAF2: TNF receptor-associated factor 2
TRAIL: TNF-related apoptosis-inducing ligand
TrkB: Tropomyosin-related kinase B
TSC1/2: Tuberous sclerosis complex 1/2
UBA: Ubiquitin-associated domain
ULK1: Unc-51-like kinase 1
UVRAG: UV radiation resistance-associated gene
VDAC: Voltage-dependent anion channel
VECs: Vascular endothelial cells
VEGF: Vascular endothelial growth factor
Vps34: Vacuolar protein sorting 34
WHO: World Health Organization
XIAP: X-linked inhibitor of apoptosis protein
