# Correction: Naturally derived indole alkaloids targeting regulated cell death (RCD) for cancer therapy: from molecular mechanisms to potential therapeutic targets

**DOI:** 10.1186/s13045-022-01371-8

**Published:** 2022-10-24

**Authors:** Rui Qin, Feng‑Ming You, Qian Zhao, Xin Xie, Cheng Peng, Gu Zhan, Bo Han

**Affiliations:** 1 State Key Laboratory of Southwestern Chinese Medicine Resources, Hospital of Chengdu University of Traditional Chinese Medicine, School of Pharmacy, Chengdu University of Traditional Chinese Medicine, Chengdu, 611137 China; 2 School of Basic Medical Sciences, Chengdu University of Traditional Chinese Medicine, Chengdu, 611137 China; 3 College of Medical Technology, Chengdu University of Traditional Chinese Medicine, Chengdu, 611137 China

## Correction to: Journal of Hematology & Oncology (2022) 15:133 https://doi.org/10.1186/s13045-022-01350-z

The original article [1] mistakenly omitted co-corresponding authorship of co-author, Gu Zhan. This has since been restored.
